# Interannual variability of the frequency of MJO phases and its association with two types of ENSO

**DOI:** 10.1038/s41598-021-91060-2

**Published:** 2021-06-02

**Authors:** Panini Dasgupta, M. K. Roxy, Rajib Chattopadhyay, C. V. Naidu, Abirlal Metya

**Affiliations:** 1 Indian Institute of Tropical Meteorology, Ministry of Earth Sciences, Pune, 411008 India; 2 Department of Meteorology and Oceanography, College of Science and Technology, Andhra University, Visakhapatnam, Andhra Pradesh 530003 India; 3 India Meteorological Department, Ministry of Earth Sciences , Pune, 411005 India; 4 Department of Atmospheric and Space Sciences, Savitribai Phule Pune University, Pune, 411007 India

**Keywords:** Atmospheric science, Climate change, Ocean sciences

## Abstract

In this study, we reexamine the effect of two types of El Niño Southern Oscillation (ENSO) modes on Madden Julian Oscillation (MJO) activity in terms of the frequency of MJO phases. Evaluating all-season data, we identify two dominant zonal patterns of MJO frequency exhibiting prominent interannual variability. These patterns are structurally similar to the Wheeler and Hendon (Mon. Weather Rev. 132:1917–1932, 2004) RMM1 and RMM2 spatial patterns. The first pattern explains a higher frequency of MJO activity over the Maritime Continent and a lower frequency over the central Pacific Ocean and the western Indian Ocean, or vice versa. The second pattern is associated with a higher frequency of MJO active days over the eastern Indian Ocean and a lower frequency over the western Pacific, or vice versa. We find that these two types of MJO frequency patterns are related to the central Pacific and eastern Pacific ENSO modes. From the positive to the negative ENSO (central Pacific or eastern Pacific) phases, the respective MJO frequency patterns change their sign. The MJO frequency patterns are the lag response of the underlying ocean state. The coupling between ocean and atmosphere is exceedingly complex. The first MJO frequency pattern is most prominent during the negative central-Pacific (CP-type) ENSO phases (specifically during September–November and December-February seasons). The second MJO frequency pattern is most evident during the positive eastern-Pacific (EP-type) ENSO phases (specifically during March–May, June–August and September–November). Different zonal circulation patterns during CP-type and EP-type ENSO phases alter the mean moisture distribution throughout the tropics. The horizontal convergence of mean background moisture through intraseasonal winds are responsible for the MJO frequency anomalies during the two types of ENSO phases. The results here show how the MJO activity gets modulated on a regional scale in the presence of two types of ENSO events and can be useful in anticipating the seasonal MJO conditions from a predicted ENSO state.

## Introduction

The Madden Julian Oscillation (MJO) and El Niño Southern Oscillation (ENSO) are the two leading modes in intraseasonal and interannual time scales in the tropics, and hence of immense importance to the global climate variability^1–4^. MJO is one of the strongest modes at intraseasonal time scale. The MJO is evident by the slow eastward propagating tropical convective branch, which usually originates over the western Indian Ocean and dies out over the cold sea surface temperature (SST) beyond the dateline. Based on outgoing longwave radiation (OLR) and zonal wind at 850 hPa and 200 hPa, Wheeler and Hendon^5^ derived a Real-time Multivariate MJO index (RMM) to track the dynamical and convective signal of the MJO. RMM index is computed from the combined empirical orthogonal functions (combined EOF) of OLR and zonal winds at 850 and 200 hPa. The eight phases of the RMM index represent the location of active and suppressed convection of the MJO and related upper and lower tropospheric circulation patterns. The major strength of the RMM index is its real-time operational use as it does not need any time filtering.

The RMM index has been extensively used to explore the role of the MJO in various weather and climate phenomena. Despite its extensive use in MJO related research and operational purposes, the RMM index has its specific limitations. By design, the RMM index is more governed by the dynamical signal (zonal winds) than the convective signal (OLR) of MJO^6^. Therefore, the use of RMM index is constricting when the research is particularly focused on MJO convection. Also, in boreal summer, the RMM index sometimes fails to isolate the MJO signal from the northward propagating intraseasonal oscillation (ISO) signal^7^. These limitations must be taken into account while using the RMM index in climate studies.

The MJO possesses profound seasonal characteristics. MJO is strongest during boreal winter and spring (December–anuary–February and March–April–May) and weakest during boreal summer and autumn (June–July–August and September–October–November)^8–10^. Lafleur et al.^10^ provided an extensive review of the seasonality of the MJO using the RMM index. The number of MJO events also varies according to seasons. Location of the MJO signal shifts southward and northward in boreal winter and summer, respectively^11,12^.

Despite belonging to widely separated time scales, the MJO and ENSO have similarities in large scale convection and circulation patterns. MJO circulation can be considered as a travelling Walker circulation pattern, while ENSO is the standing pattern. Similarities between MJO and ENSO have led to extensive studies on their relationship in the past few decades^13–16^. These studies suggest that the mature El Niño or La Niña phase expands the region of MJO activity further eastward or westward^17^. The inter-annual variation of MJO is therefore observed along the boundaries of the intra-seasonal variability center, the Indo-Pacific warm pool. In fact, the Indo-Pacific warm pool conditions modulate the MJO characteristics both on interannual to long-term climate time scales^18^. Besides this simultaneous connection between the MJO and ENSO, Hendon et al.^19^ observed a lag relationship between the MJO activity in spring (March–April–May) and the state of ENSO in subsequent winter (December–January–February). This explains the role of MJO in the initiation of ENSO events. Strong westerly winds at the western Pacific associated with MJO triggers the downwelling Kelvin wave^20^. As the Kelvin wave propagates eastward, the warmpool expands and develops the positive ENSO phase. Chen et al.^21^ observed that MJO activity is generally strengthened during the predeveloping phase of EP-type ENSOs, but for the CP-type ENSOs, the role of MJO is not clear. On the contrary, during the developing and mature stages, MJO activity strengthens in CP-type ENSOs, but no detectable changes are observed for EP-type ESNO. Pang et al.^22^ examined the effects of two types of ENSO, i.e., canonical and central Pacific warm-cold events on boreal winter MJO. They observed that the MJO strength is generally weaker during the canonical warm period and stronger during the canonical cold period. The case is opposite for central Pacific ENSO events where the MJO strength is generally stronger during warm phases and is weaker during central Pacific cold phases. Feng et al.^23^ suggested that during eastern Pacific ENSO events, MJO amplitude is generally weaker during MJO phases 4–6. Hsu and Xiao^24^ discussed the reason behind the strengthened MJO propagation over the Indian Ocean during central Pacific ENSO events compared to eastern Pacific ENSO events. Overall, these studies are mainly focused on the interannual variation of the strength of MJO convection or the amplitude.

Similar to the MJO amplitude, the number of MJO active days (frequency of occurrence) in a year or season also undergoes interannual variations^25^. The number of MJO active days are often related to MJO propagation speed or number of MJO events in a time span. The frequency of the MJO at different phase locations is also affected by the two ENSO types^22^. For example, the frequency of MJO phase 2,3 and 4,5 increases during EP El Niño winters and CP El Niño winters respectively^22^.

ENSO is known to have global impacts when it is not extreme. ENSO influences the other basins (the tropical Indian Ocean and Atlantic Ocean) through tropospheric Kelvin and Rossby waves and the anomalous Walker circulation. ENSO affects the mid-latitude and higher latitude weather system through the stationary wave train originating from the extratropical heat source and eddy-jet stream mechanism^26^. Similar to ENSO, the MJO also exhibits large scale teleconnection patterns as it propagates eastward. MJO teleconnection patterns in phase 3 and 7 are similar to the positive and negative ENSO teleconnection patterns^27^. During the ENSO phases, the superposition of ENSO and MJO teleconnection pattern affects the overall teleconnection^17^. Recent studies suggest that the frequency of MJO activity over the Indian Ocean and western Pacific can influence North Atlantic Oscillation (NAO), Pacific North America pattern (PNA) and Atlantic Meridional Oscillation (AMO) through teleconnections^28^. The frequency of MJO activity, therefore, is an important factor to be understood properly to comprehend the MJO teleconnections.

In most of the earlier studies, the inter-annual variability of MJO and its relation to ENSO were mainly investigated by examining any particular season i.e. boreal winter, spring or summer^28,29^. ENSO evolution, however, generally starts from boreal spring (March–April–May), matures during boreal winter (December–January–February) and decays during the subsequent boreal spring^30^. Therefore, it is important to examine interannual variability of MJO and its relation to ENSO considering all the seasons (throughout the year). Secondly, previous studies majorly focused on the intensity of MJO (variance in intra-seasonal OLR) for studying the inter-annual variability of the MJO. However, recent studies^18,25,28,31^ demonstrate that interannual variability of the MJO is more evident in the variation in the number of days MJO convection spent over the certain region (i.e. the frequency of occurrences of MJO phases) rather than its amplitude. The signature of MJO phase occurrence in a season is evident through the seasonal mean convective activity over the respective phase regions. In the present study, we examined the inter-annual variability of the MJO in terms of the frequency of occurrence of the MJO phases, where we consider MJO activity in all four conventional seasons (throughout the year). The major goal of the present study is to find dominant spatial modes of MJO variability. We have employed multivariate Principal Component Analysis (PCA) and PCA Biplot to visualize and interpret our results. Biplot technique to visualize the PCA result is not very common in meteorology. We have tried to implement this method for examining the inter-annual variability of MJO phase frequency.

## Results

### Dominant Modes of interannual variability of MJO Frequency anomaly

**Figure 1:**
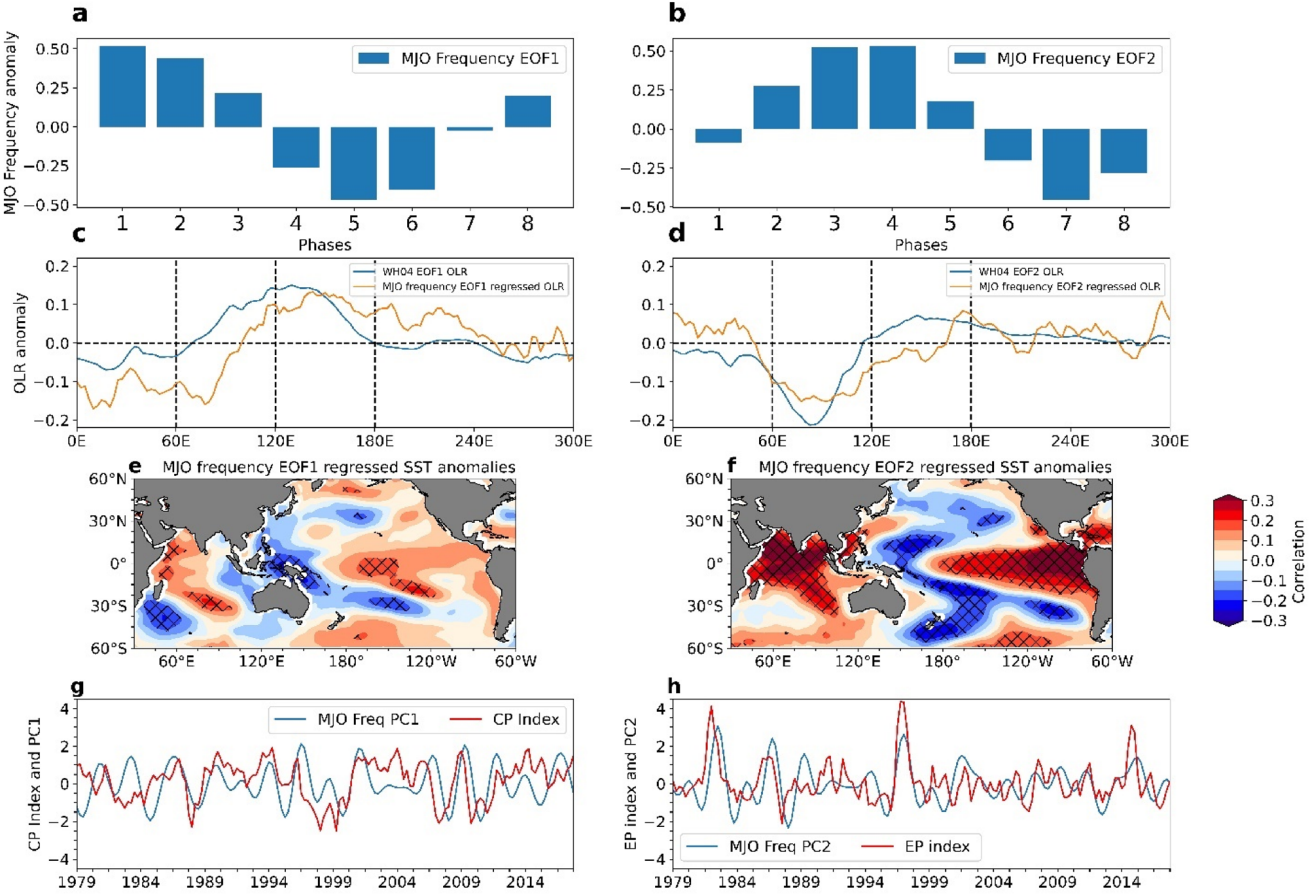
(**a**,**b**) First two leading spatial EOFs of MJO frequency variability (days). (**c,d**) Original WH04 RMM1 and RMM2 spatial pattern (blue lines) and spatial pattern of MJO frequency EOF1 and EOF2 of OLR. (**e,f**) SST anomalies related to MJO frequency EOF1 and EOF2 (hatched denote the locations having significant correlation with 90% confidence level). (**g**) represents PC1 timeseries (2–8 years bandpass filtered) (blue line) and CP El Niño index (red line). (**h**) shows PC2 timeseries (2–8 years bandpass filtered) (black line) and EP El Niño index (red line).

Eight phase locations of MJO depict the different sections of the tropics through which the MJO propagates. Phase regions 1 to 3 represent west to central tropical Indian Ocean. Phase regions 4 and 5 depict the eastern Indian Ocean and Maritime Continent. Phases 6 to 8 represent west to central tropical Pacific region. MJO frequency anomalies at the eight MJO phase locations in DJF, MAM, JJA and SON explain the spatial variation of MJO activity throughout the year (Supplementary Fig. S1a). In this study, we identify the spatial modes of MJO frequency variation by employing EOF analysis on MJO frequency anomaly (Fig. 1a,b). Supplementary Figure S1b represents the percentage of variance explained by the eight EOFs of MJO frequency anomaly. It is important to note that the first two EOFs explain almost half (48%) of the total variance in MJO frequency anomaly (Fig. 1 a,b). EOF1 and EOF2 explain about 25.7% and 22.2% of the total variance. The remaining EOFs explain the rest of the variability with nearly equal contributions. The first two EOFs are significantly separated from each other and rest of the EOFs according to North et al.^32^ criteria. We find that MJO frequency pattern associated with the first two EOFs do not change appreciably over time (Supplementary Fig. S2). EOF1 represents an out of phase pattern of MJO frequency anomaly between the phase regions 1, 2, 8 and 4, 5, 6 (Fig. 1a). This means that a positive MJO frequency anomaly at phase regions 1, 2, and 8 (west to central Indian Ocean) coincide with negative (opposite) MJO frequency anomaly in phase regions 4, 5 and 6 (eastern Indian Ocean to the Maritime Continent to the western Pacific). The MJO frequency anomaly variations at phase 3 (central Indian Ocean) and 7 regions (west-central Pacific) are less in this variation pattern. Phase 1 (western Indian Ocean) and 5 (Maritime Continent) MJO frequency variations are most dominant in EOF1 pattern. The structure of MJO frequency EOF1 pattern is similar to RMM1 spatial pattern (Fig. 1c). In Fig. 1c, we represent the original WH04 EOF1 OLR and MJO frequency EOF1 related OLR pattern (zonal mean). We observe that the MJO frequency EOF1 structure is similar to the WH04 RMM1. The second EOF also denotes a similar out-of-phase relationship of MJO frequency anomaly between phase regions 2, 3, 4 (central to east Indian Ocean) and 6, 7, 8 (west to central Pacific) (Fig. 1b). In the second EOF structure, MJO frequency anomaly is small over phase regions 1, 5 (western Indian Ocean and Maritime Continent) and are mostly large in phase regions 3, 7 (central Indian Ocean and western Pacific). Similarly, as the MJO frequency EOF1, MJO frequency EOF2 pattern is similar to WH04 RMM2 spatial pattern (Fig. 1d). We calculated the correlation between three monthly mean (DJF, MAM, JJA, SON) WH04 RMM1 timeseries and MJO frequency EOF1 time-series and three-monthly mean WH04 RMM2 timeseries and MJO frequency EOF2 time-series where we obtained 0.74 and 0.68 correlations. These correlations suggest that interannual variation of MJO activity follows the WH04 RMM spatial patterns.

Interannual variability of MJO frequency at the eight phase locations and their inter-relationship, can be visualized in a two-dimensional plane formed by the two leading MJO frequency EOFs through the biplot technique (Supplementary Fig. S3). We discussed the details of biplot in the method section. MJO frequency anomaly variation at the eight phase locations are represented by the eight arrows in the biplot. The length of an arrow represents the variance of MJO frequency in a certain phase location explained by the two leading EOFs (Supplementary Fig. S3). An arrow’s direction depends on the correlation of MJO frequency variation at a location with two EOF time series. It tells the extent to which the frequency variation in a certain phase is closely related to either of the spatial modes. As we can see, MJO frequency variation at phase locations 1 and 5 are closely related to EOF1 spatial modes. Similarly, MJO frequency variation at phase regions 3 and 7 are related to EOF2 spatial modes. The MJO frequency variations at these phase locations provide maximum variance to the MJO frequency spatial modes. The arrows which group together by having the same direction in the two-dimensional plane, represent the positively correlated MJO phase locations having similar MJO frequency variations. The cosine of the angle between two arrows denotes the correlation among MJO frequency in the corresponding locations. The in-between relationship among the MJO frequencies at 8 phase locations suggests that a positive MJO frequency anomaly in a particular phase region is also associated with the negative MJO frequency anomaly at the opposite phase quadrant in the RMM phase diagram. This is because in the RMM phase diagram, MJO is represented by wavenumber 1 pattern in the tropical belt^33,34^.

We obtained correlation coefficients and cosine square, two statistical quantities, to quantify the goodness of representation of the MJO frequency variation through the leading EOFs. The cosine square parameter represents the percentage of the variance of MJO frequency over a location expressed through a certain EOF (Supplementary Fig. S3). The sum of cosine square values for all the EOFs is equal to 1. We also measured the correlation coefficient between MJO frequency time series at eight phase locations and the EOF time series to obtain the goodness of representation of MJO frequency data by the two leading EOFs. In Supplementary Table S1 the correlation coefficient and cosine square values are presented. The significant correlation value for 156 data points at 99% confidence level is 0.21. Considering the cosine square and correlation coefficient, we observed that EOF1 well represents the MJO frequency variation at phase locations 1, 2, 5, and 6 (where correlation values exceeds 0.5). Similarly, EOF2 represents the MJO frequency variation in phase 3, 4, 7 and 8 (Supplementary Table S1). From correlation coefficients and cosine square values, it becomes reasonable to study the MJO frequency variations in terms of these two spatial EOF modes. MJO frequency in terms of two leading EOF modes also is a dimension reduced form (8 dimensions to 2 dimensions) of MJO frequency anomaly data which is easier to be analyzed than higher dimensional data.

To visualize the MJO frequency modes in terms of intraseasonal convection (OLR), we obtained correlated filtered OLR (20–100 days filtered) pattern of MJO frequency EOF1 and EOF2. (Supplementary Fig. S4 a,b). MJO frequency EOF time-series has four data points per year (DJF, MAM, JJA, SON); from 1979–2018 total of 156 data points. We regressed the two EOF timeseries with three monthly mean OLR/precipitation anomalies (daily) to extract the spatial patterns. MJO frequency anomaly represents the change in the number of MJO convective days. A change in the number of convective days in a season is generally expected to be evident in seasonal mean OLR rather than the variance of the OLR in that season. The variance of filtered refers to the strength or amplitude of MJO convection, whereas a negative mean of filtered OLR expresses the fact that a significant part of the signal represented the negative OLR anomaly due to a higher number of convective days. We obtained the correlated MJO precipitation (space–time filtered) anomaly associated with MJO frequency EOF1 and EOF2 in Supplementary Figure. S4c,d. The filtered OLR and precipitation pattern of EOF1 (Supplementary Fig. S4a,c) shows wet western Indian Ocean and central-east Pacific, when the Maritime Continent and western Pacific regions are dry, and vice versa. The filtered OLR and precipitation pattern for the second mode denotes a wet eastern Indian Ocean when the western Pacific is dry and vice versa (Supplementary Fig. S4b,d).

### MJO frequency EOFs and its relationship with seasonal mean SST anomaly

**Figure 2:**
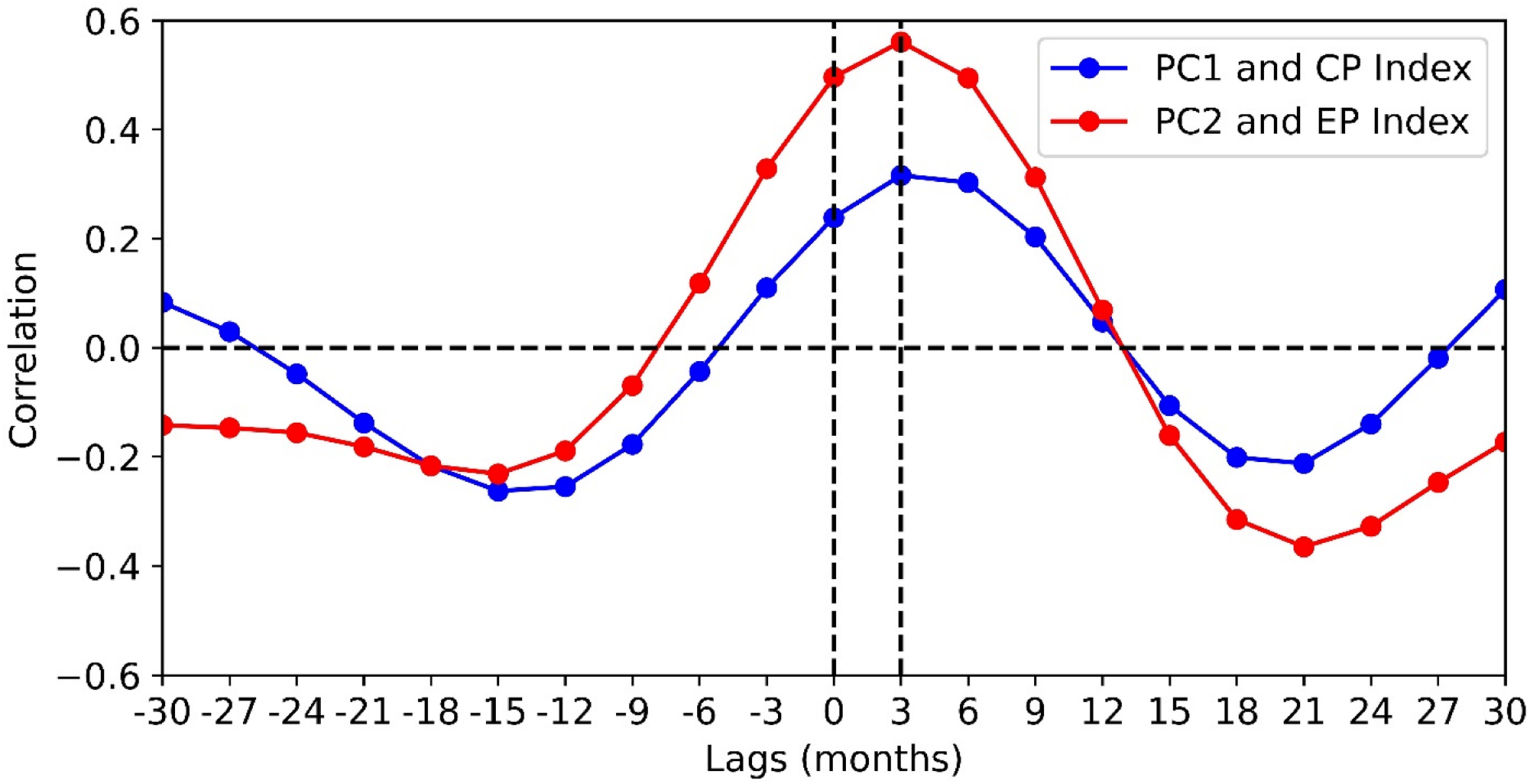
Lead-lag relationship between PC1 timeseries (2–8 years bandpass filtered) and CP ENSO index (blue line). It is observed that negative CP El Niño leads PC1 timeseries by one season with correlation value 0.31. At lag 0 correlation is approximately 0.24. Lead-lag relationship between PC2 time series (2–8 years bandpass filtered) and EP ENSO index (blue line). EP ENSO index leads PC2 timeseries by one season with correlation value 0.56. At lag 0 correlation is approximately 0.5.

To examine the mean state of the tropical ocean associated with the MJO frequency modes, we obtained the correlated seasonal mean SST anomalies of EOF1 and EOF2 time series (Fig. 1e,f). We observed that the first MJO frequency mode (EOF1) is apparently related to central Pacific warm event structure or El Niño Modoki structure (Fig. 1e, Ashok et al.^35^). The correlation between PC1 (2–8 years filtered) and CP ENSO index is significant at 95% confidence level with a correlation value 0.24 (Fig. 1g). The associated SST pattern with the second MJO frequency mode (EOF2) shows an EP-type canonical ENSO pattern. The correlation between PC2 (2–8 years filtered) and EP ENSO index is significant at 95% confidence level with correlation value 0.47 (Fig. 1h). We further examined the lead-lag correlation between PC1 (2–8 years filtered) and CP ENSO index, where we found that the CP ENSO index leads PC1 MJO frequency pattern by one season (3 months) with peak correlation value 0.3. At lag 0 the correlation is approximately 0.24 (Fig. 2). From the lead-lag relationship between PC2 time series (2–8 years bandpass filtered) and EP ENSO index, we found that the EP ENSO index leads PC2 timeseries by one season with peak correlation value 0.57 (Fig. 2). At lag 0 the correlation is approximately 0.49. Apparently, these MJO frequency patterns fully establish in response to the underlying ocean condition at a 3 months lag. The reason for the weak correlation between PC1 (2–8 years filtered) and CP ENSO index may be due to the sample size in our present study. ENSO is a 2–8 years event and the number of ENSO events during 1979–2018 are few. Generally, the SST variability in the central Pacific is not as large as in the east Pacific, which may also have a bearing on the correlation. The time series of PC1 (2–8 years filtered), CP ENSO index and PC2, EP ENSO index are represented in Fig. 1g,h. These time series plots show significant associations between two MJO frequency modes and two ENSO types.

Since a correlation analysis may not clearly represent the relationship between MJO frequency modes and two ENSO types, we have analyzed the composites of CP-type and EP-type El Niño/La Niña years in the next section.

### Composites of MJO frequency during canonical and Central Pacific warm and cold events

**Figure 3:**
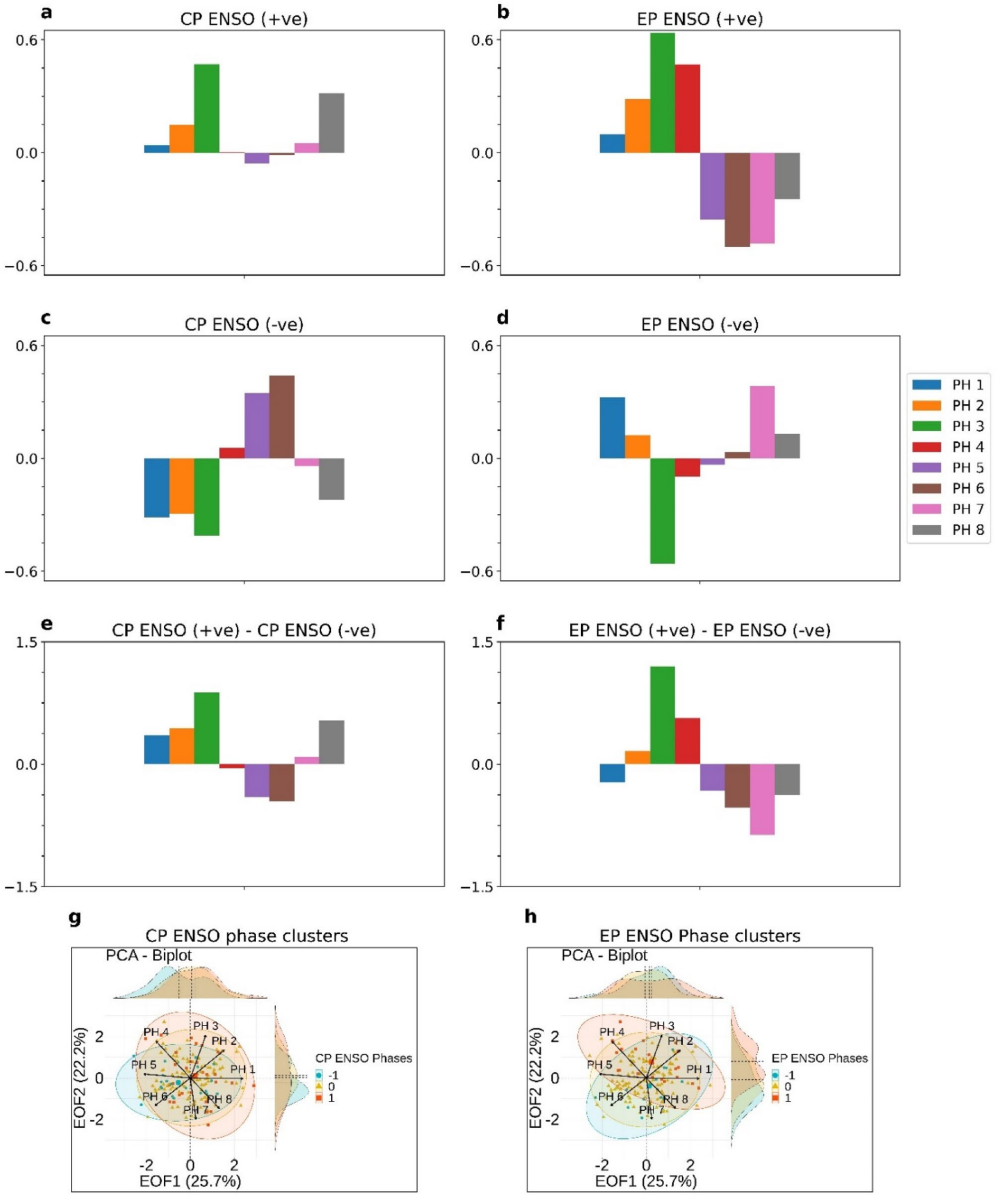
Composites of MJO frequency anomalies (standardized) at 8 RMM phase regions during (**a**) Positive CP-type ENSO seasons, (**c**) Negative CP-type ENSO seasons and their differences (**e**). Similarly, composites for (**b**) Positive EP-type ENSO seasons, (**d**) Negative EP-type ENSO seasons and their differences (**f**). (**g**) PCA biplot and clusters of positive (red), negative (blue) and neutral (yellow) CP-type ENSO seasons. Data ellipses represent the statistics of different classes. (**h**) Similar as the figure (**g**) for positive, negative and neutral EP-type ENSO phases.

The association between two types of ENSO modes and MJO frequency modes are further examined through a composite analysis. Composites of MJO frequency anomaly during EP-type canonical warm/cold and central Pacific warm/cold seasons are prepared. If the modes are associated with two respective types of ENSO, then composites of frequency anomaly during these ENSO phases should match with the EOF MJO frequency patterns. We observed the MJO frequency composites during warm and cold CP-type ENSO phases and their composite differences resemble EOF1 MJO frequency pattern (Fig. 3a,c,e). Similarly, MJO frequency composites during warm and cold EP-type canonical ENSO events and their differences resemble EOF2 MJO frequency pattern (Fig. 3b,d,f). These composites reveal a possible association between two frequency modes and two types of ENSO events. Importantly, we see that MJO frequency EOF1 pattern is most prominent during cold CP-type ENSO phases (Fig. 3c), and MJO frequency EOF2 pattern is most prominent during warm EP-type ENSO phases (Fig. 3b).

### MJO frequency variation through biplot and clusters

The relation between MJO frequency and ENSO phases are explored from a different perspective using the biplot technique. The biplot represents the 156 seasons of MJO frequency in a two-dimensional plane where MJO frequency EOFs are the axes (Fig. 3g,h). The MJO frequency in a particular season is represented by a point where its location is estimated by the standardized amplitude of EOF time series. The points in a biplot basically represent a scatterplot in two dimensional EOF plane. MJO frequency points which make a cluster in the biplot space have similar projections on two EOF modes which denotes the fact that seasons had similar MJO frequency anomalies. Distance between two points in biplot space is called Mahalanobis distance^36^, indicating the statistical similarity between the points representing MJO events.

From 1979 to 2018, all seasons are divided into three classes according to CP and EP ENSO index (Supplementary Fig. S5) representing the warm, cold and neutral central Pacific CP-type and canonical EP-type ENSO phases. The points representing a particular class is enclosed by the data ellipse which explains the overall statistics of the points (Fig. 3g,h) in the biplot space. The joint distributions of standardized principal components i.e. PC1 and PC2 for three classes are also represented in Fig. 3g,h. The mean, standard deviation and correlation of PC1 and PC2 for different classes are represented in Table1.

**Table 1:** Mean, standard deviation of normalized PC1 and PC2 in different ENSO classes.

Variables	EOF1 (PC1)		EOF2 (PC2)	
	Mean	STD	Mean	STD
EP—El Niño	0.23	1.18	0.80	0.88
EP—La Niña	0.15	0.96	− 0.40	0.92
CP—El Niño	0.20	0.98	0.11	1.21
CP—La Niña	− 0.52	1.00	− 0.22	0.72

In Fig. 3g, data ellipses represent warm, cold and neutral CP-type ENSO phases. It is observed from the Fig. 3g that from cold to warm CP-type ENSO phases, the center (mean state) of the data ellipse shifts from negative to positive EOF1 axis. This fact is also evident from the probability distribution of EOF1 amplitude during positive, negative and neutral CP-type ENSO phases. It can be seen that the mean of distribution shift from negative EOF1 axis to positive EOF1 axis. This means that positive MJO frequency anomalies are seen at MJO phase regions 1, 2 and 8 (the western Indian Ocean and central Pacific) and negative MJO frequency anomalies are seen at phase regions 5 and 6 (Maritime continent) during warm central Pacific ENSO phases. This MJO frequency anomaly pattern gets reversed during the negative phase of central Pacific ENSO.

Similar to CP-type ENSO phases, three clusters of EP-type ENSO phases are presented in Fig. 3h. In contrast to the case of CP-type ENSO phases, the center of the data ellipse shift from negative EOF2 axis to positive EOF2 axis during canonical EP-type ENSO negative to a positive phase. The shifts in data ellipses centers are associated with the mean MJO frequency pattern changes during cold to warm EP-type ENSO phases. In the case of EP-type ENSO phases, overall MJO frequency changes following the EOF2 axis. This means that positive MJO frequency anomalies are seen at MJO phase regions 3 and 4 (central-east Indian Ocean) and negative MJO frequency anomalies are seen at phase regions 6 and 7 (west to central Pacific) during warm canonical ENSO period. This MJO frequency anomaly pattern gets reversed during the negative EP-type ENSO period. The warm and cold EP-type ENSO data ellipse has directions along with MJO phases 4, 8 and MJO phases 2, 6, respectively. This means that during the canonical warm period, MJO frequencies in phase regions 4 and 8 have the highest variability. Either of the phase region 4 (eastern Indian Ocean) or phase region 8 (central Pacific) may have large MJO frequency anomaly. Similarly, during the canonical La Niña, either of the phase region 2 (central Indian Ocean) or phase region 6 (western Pacific) may have large MJO frequency anomaly.

Therefore, the shift of an ellipse center from cold to warm phases of ENSO modes indicates the basic state change of MJO frequency pattern. These basic state changes in the MJO frequency pattern associated with the ENSO modes are the possible reasons behind the observed correlation between MJO frequency EOFs and two type ENSO indices. It is important to note that time series of two modes of MJO variability follows normal distribution at a 99% confidence level as evident in D’Agostino and Pearson’s test.

### Composite differences between central Pacific and eastern Pacific warm and cold events

**Figure 4:**
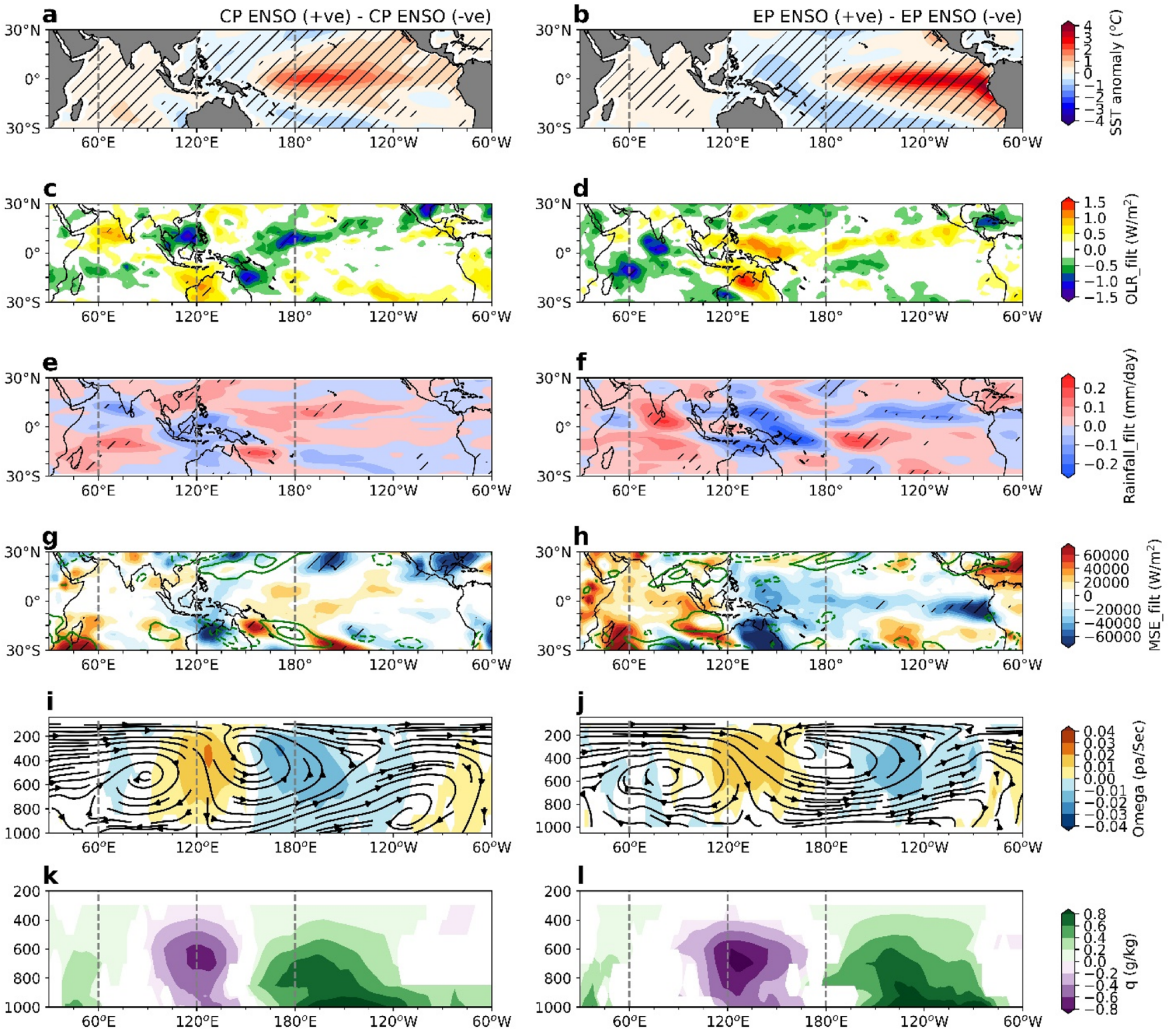
Composite differences of different fields between positive and negative CP-type and EP-type ENSO phases. (**a,c,e,g,i**,**k**) represent the composite differences of SST, 20–100 days filtered OLR, space–time filtered precipitation, 20–100 days filtered vertically integrated moist static energy anomaly $\left\langle {m^{{\prime }} } \right\rangle$ and moist static energy tendency $\left\langle {dm^{{\prime }} /dt} \right\rangle$, omega and specific humidity respectively. Similarly, (**b,d,f,h,j, l**) represent the composite differences of specified fields between positive and negative EP-type ENSO seasons. Hatches represents the 90% confidence level. In (**i,j,k,l**) only anomalies exceeding 90% confidence level are plotted.

The composite differences of SST, filtered OLR, space–time filtered precipitation, vertically integrated MSE, MSE tendency, zonally averaged circulation and specific humidity between the warm and cold phases of the CP-type ENSO are represented in Fig. 4a,c,e,g,i,k. Similarly, composite differences for EP-type ENSO are represented in Fig. 4b,d,f,h,j,l. We derived the composite differences of different parameters between the warm and cold phases of ENSO to investigate the reason for basic state shift of MJO activity following the MJO frequency EOF patterns.

For the CP-type ENSO, the composite differences between warm and cold phases indicate the enhanced intraseasonal precipitation at the western Indian Ocean and central Pacific (RMM phase 1,2 and 8) and suppressed intraseasonal precipitation over the maritime continent (RMM phase 5 and 6) which is similar with the MJO frequency EOF1 pattern (Fig. 4a,c,e). The outcome from the biplot of CP-type ENSO phases also suggest the mean state change following the MJO frequency EOF1 pattern. For the EP-type ENSO, the composite differences between warm and cold phases show the enhanced intraseasonal rainfall near eastern equatorial Indian Ocean and maritime Continent and suppressed intraseasonal rainfall over west-central Pacific Ocean. This is also consistent with the MJO frequency EOF2 pattern and biplot outcome for EP-type ENSO phases (Fig. 4b,d,f).

To investigate the reason for the particular MJO frequency anomaly patterns associated with the two types ENSO phases, we examined the composite differences of vertically integrated intraseasonal MSE anomalies, vertically integrated intraseasonal MSE tendencies, zonally averaged circulation and specific humidity profiles in Fig. 4g–l. For CP-type ENSO phases there are negative intraseasonal MSE anomalies over the maritime continent (RMM phases 5 and 6) and positive MSE anomalies over the west and central Indian Ocean (RMM phases 1and 3) and Central Pacific (RMM phase 8) (Fig. 4g). The negative tendency of intra-seasonal MSE is also observed over the maritime continent (the same region with negative MSE anomalies) decreasing the lifetime of MJO over the maritime continent. The positive MSE tendency is observed over the west-central Indian Ocean and central Pacific enhancing the MJO lifetime over these regions. We find that the mean zonal circulation favors the convection over the western Indian Ocean and the central Pacific Ocean and opposes convection over the Maritime Continent during the CP-type ENSO events (Fig. 4i). During these times, the main descending branch of the Walker circulation is situated over the eastern equatorial Indian Ocean and maritime continent centering at 120°E. The mean moisture distribution (specific humidity) over the maritime continent shows the negative anomalies centered at 120°E following the zonal circulation pattern (Fig. 4k). Therefore, the mean background moisture distribution is a vital factor determining the intraseasonal MSE tendency. The intraseasonal MSE tendency term generally depends on the advection of mean background moisture by intraseasonal easterly winds. For the CP-type ENSO seasons, negative background moisture anomalies over the Maritime Continent creates negative intraseasonal MSE tendency which restricts the MJO propagation over these regions.

In the case of canonical EP-type ENSO phases, the main descending branch of the Walker circulation is situated over the maritime continent and western Pacific at the east of 120°E (Fig. 4j). The mean moisture distribution (specific humidity) over the maritime continent and west Pacific shows the negative anomalies at the east of 120°E following the zonal circulation pattern (Fig. 4l). Therefore, the intraseasonal MSE tendency term is negative over the maritime continent and west Pacific, restricting the MJO propagation over these regions (Fig. 4h). The intra-seasonal MSE tendency over the eastern Indian ocean is positive due to the moisture contribution from western pacific surface divergence. Vertically integrated intraseasonal MSE anomalies are positive over the central and eastern Indian Ocean and negative at the east of 120°E following the intra-seasonal MSE tendency pattern. Therefore, during the EP-type ENSO phases, positive intraseasonal precipitation anomalies are observed at central and eastern equatorial the Indian Ocean and negative intraseasonal precipitation anomalies are observed over the western and central Pacific.

**Figure 5:**
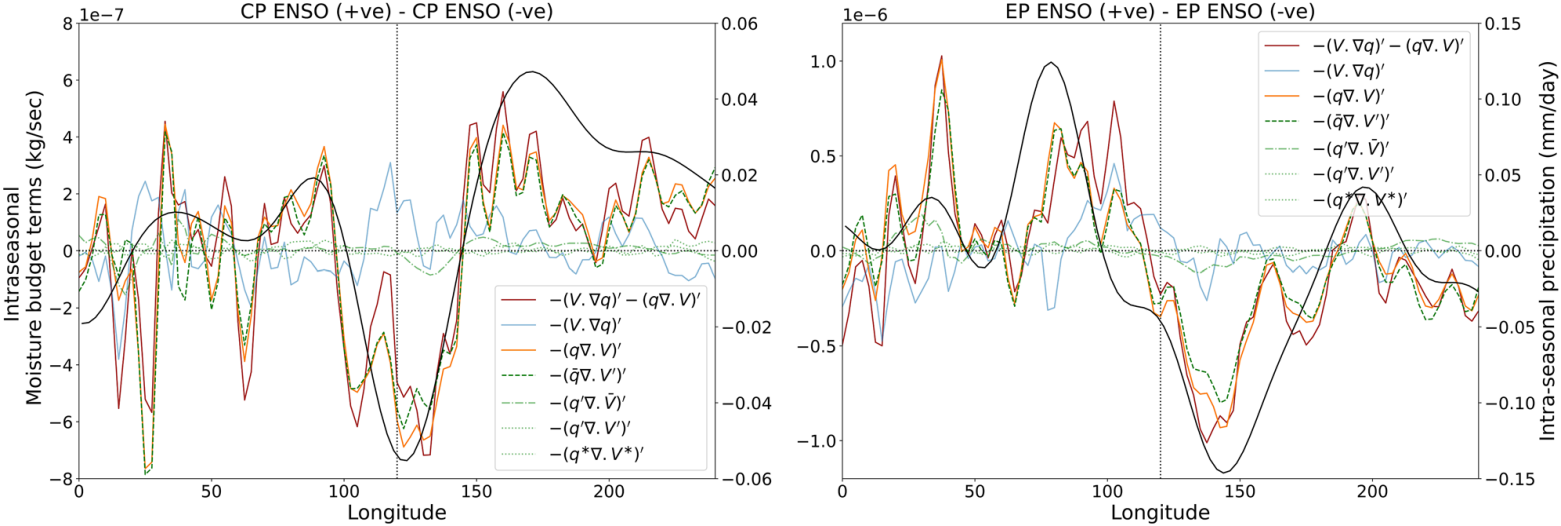
Differences in moisture budget terms in Eqs. (2 and 4) between the warm and cold CP-type ENSO phases (left panel). The red line denotes the horizontal moisture flux convergence [sum of horizontal advection (blue) and moisture convergence (yellow)]. The green lines are the four terms of horizontal moisture convergence in Eq. (4). We observed that the dashed green line denoting the convergence of mean moisture through intraseasonal winds have the largest contribution in intra seasonal moisture tendencies (vertical advection due to horizontal moisture convergence). The right panel shows the difference in moisture budget terms for warm and cold EP-type ENSO phases.

We further investigated how the mean moisture distribution alters the intraseasonal precipitation. Intraseasonal convection occurs due to the moisture tendency at intraseasonal time scale. We conducted intraseasonal moisture budget analysis to inspect the contributor of moisture tendency mentioned in Eq. (2). Details about the vertically integrated intraseasonal moisture budget is described in the method section. It is observed that intraseasonal moisture tendency is governed by the horizontal and vertical advection of moisture (terms 1–3 in RHS of Eq. (2)). The vertical advection of moisture is governed by horizontal moisture convergence $- \left( {q\nabla \cdot V} \right)^{{\prime }}$. We investigated the moisture tendency source during CP and EP-type ENSO phases. We computed the composite difference of horizontal moisture advection $- \left( {V \cdot \nabla q} \right)^{{\prime }}$ and horizontal moisture convergence $- \left( {q\nabla \cdot V} \right)^{{\prime }}$, between the warm and cold CP-type (Fig. 5 left panel) and EP-type ENSO phases (Fig. 5 right panel) to investigate the source of moisture tendency. We observe that the moisture tendency is largely governed by horizontal moisture convergence denoted by $- \left( {q\nabla \cdot V} \right)^{{\prime }}$. Interestingly, the zonal distribution of horizontal moisture convergence (vertically integrated) has tripolar structure during CP-type ENSO phase and dipolar structure during EP-type ENSO phases (similar to MJO frequency patterns/WH04 RMM spatial patterns) following the mean intraseasonal precipitation distribution. This fact suggests that the horizontal moisture convergence is the key reason for the intraseasonal precipitation anomalies during the two types of ENSO. The horizontal moisture convergence term further can be decomposed into four parts according to Eq. (4). In Eq. (4), there are four terms i.e., $- { }\left( {\overline{q}\nabla \cdot V^{{\prime }} } \right)^{{\prime }} , - \left( {q^{{\prime }} \nabla \cdot \overline{V}} \right)^{{\prime }} , - \left( {q^{{\prime }} \nabla \cdot V^{{\prime }} } \right)^{{\prime }} , - \left( {q^{*} \nabla \cdot V^{*} } \right)^{{\prime }}$. We investigated the contribution of these four terms to intraseasonal moisture convergence and thereby to moisture tendency. We observe that for both CP-type and EP-type ENSOs, the convergence of mean moisture due to the intraseasonal winds $- { }\left( {\overline{q}\nabla \cdot V^{{\prime }} } \right)^{{\prime }}$ plays the dominant role. In conclusion, during the CP-type and EP-type ENSO, mean moisture distribution and its convergence through intraseasonal winds are responsible for the anomalous MJO frequency pattern.

### MJO propagation during the central Pacific and eastern Pacific warm and cold events

**Figure 6:**
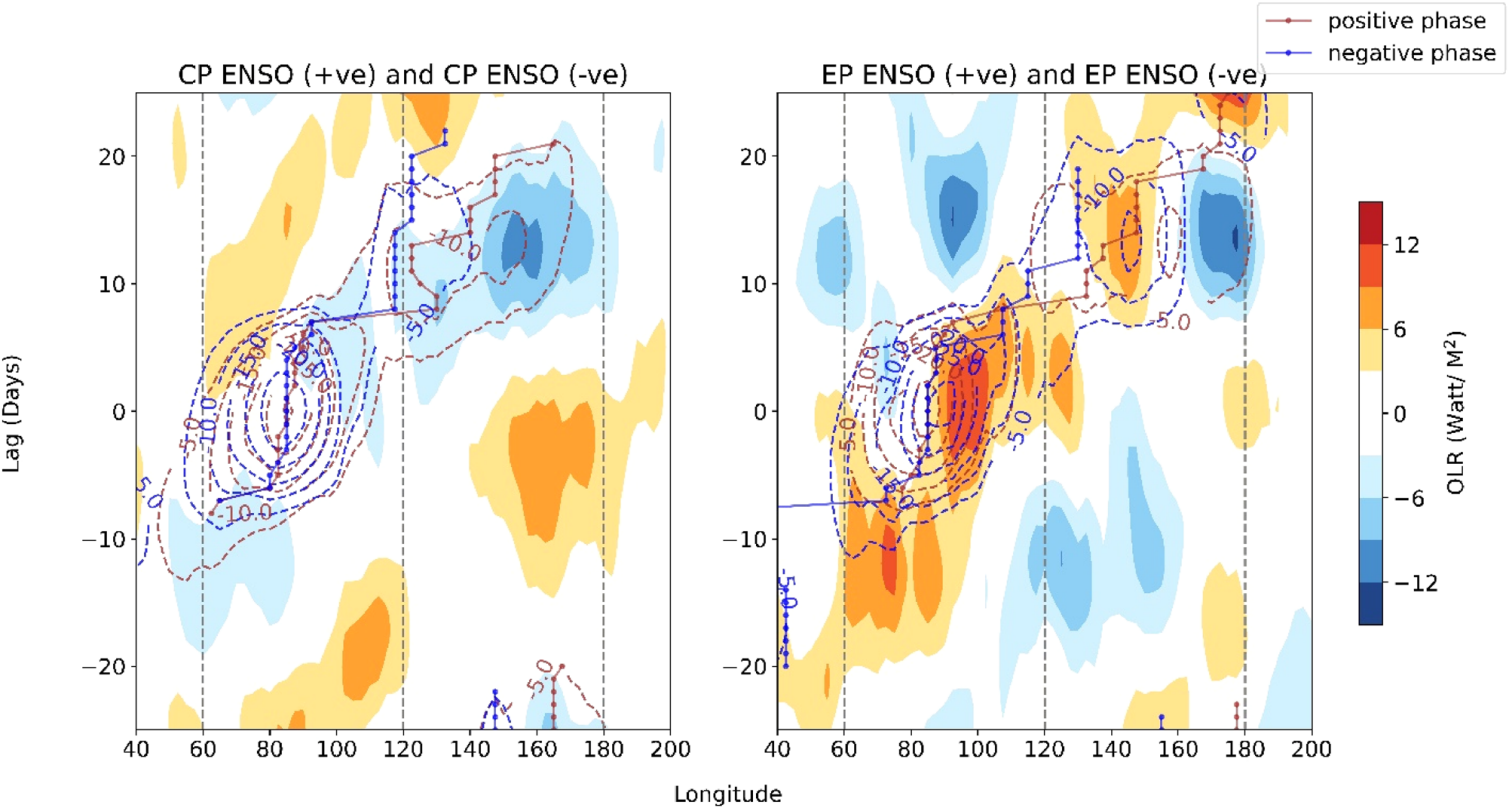
Lag propagation of 20–100 days filtered OLR (Watt/m^2^) with respect to central Indian Ocean (80°E–90°E, 15°S–15°N). Left panel shows the MJO propagation during positive (brown contour) and negative (blue contour) CP-type ENSO phases. The shade denotes the difference in MJO propagation between warm and cold phases. The dotted lines represent the location (longitude) of maximum lag-correlation with central Indian ocean at each time lag (brown: warm phase, blue: cold phase). This indicate the path of the MJO center as a function of time lags. Similarly, as the left panel, the right panel shows the MJO propagation for the positive and negative EP-type ENSO phases.

The propagation features of intraseasonal OLR during the warm and cold phases of CP-type and EP-type ENSO events are shown in Fig. 6. In Fig. 6 (left panel), lag-longitude propagations of OLR in warm (brown contours) and cold CP-type (blue contours) ENSO and their differences are represented. We observe that during the warm CP-type ENSO phases, the MJO propagates eastward up to central Pacific 180°E, whereas during the cold CP-type ENSO phases, MJO propagation is limited up to 140°E. During the cold CP-type ENSO, MJO spends more time around 120°E. The composite differences of OLR show during the warm CP-type ENSO, the western Indian Ocean and central Pacific are comparatively wet (with negative OLR indicating more vigorous convection) compared to the cold CP-type ENSO. Compared to the cold EP-type ENSO phases, MJO propagation is faster but weaker in strength during warm EP-type ENSO (Fig. 6, right panel). During the cold EP-type ENSO, the MJO spends more time over the western Pacific, whereas in the warm phase, MJO spends more time over the central Indian Ocean around 80°E. The difference in MJO strength between warm and cold EP-type ENSO point out the difference in their residence time over the central Indian Ocean and the western Pacific.

We also investigated the MJO dwelling time in different locations by computing the cross-correlation value. The location (longitude) of maximum cross-correlation (with respect to the central Indian Ocean) at each time lag is identified. These locations roughly provide an idea about how much time the MJO resides at each of the longitudes. When the maximum correlation location stays at the same longitude for consecutive timesteps, it denotes that the MJO convection is spending a long time over that particular longitude (more MJO frequency over that longitude). For the cold CP-type phases, we find that the MJO spends more time around 120°E (13 days) compared to CP-type positive phases (6 days). Similarly, for the cold EP-type phases, MJO spends more time over the maritime continent and western Pacific between 110°E–150°E (11 days) than the warm EP-type phases (5 days). During the warm EP-type phases, MJO spends more time at the central and eastern equatorial Indian ocean between the 80°E–100°E (16 days) compared to the EP-type negative phases (12 days). This representation approximately provides the differences in the MJO propagations between the warm and cold ENSO phases.

### MJO frequency anomalies during two types of ENSO phases in the RMM1-RMM2 plane

**Figure 7:**
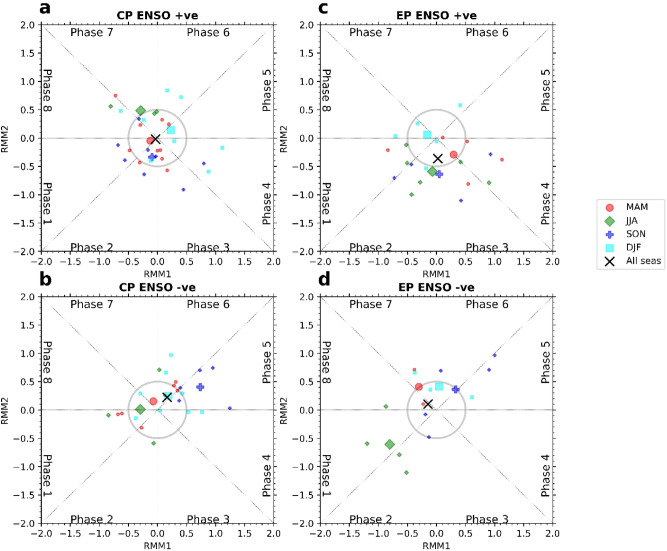
The mean location of MJO in WH04 RMM1-RMM2 plane during EP and CP-type ENSO seasons (MAM : red, JJA: green, SON: blue and DJF: cyan). (**a**,**c**) Represent the mean MJO location during positive and negative CP-type ENSO phases. Similarly, (**b**,**d**) represent the mean MJO location during positive and negative EP-type ENSO phases. The mean MJO location has been calculated from the mean of RMM1 and RMM2 value during DJF (cyan), MAM (red), JJA (green), and SON (blue) when $\sqrt {RMM1^{2} + RMM2^{2} } > 1.0$ (MJO active condition). The black cross mark represents the mean position of MJO considering all the season.

MJO frequency anomalies during two types of ENSO phases also can be visualized in the WH04 RMM1-RMM2 plane since MJO frequency EOFs (EOF1 and EOF2) are related to the WH04 RMM (RMM1 and RMM2) patterns. From cold to warm CP-type ENSO phases, MJO frequency changes following the MJO frequency EOF1 pattern (Fig. 3g). In Fig. 7a,b, we represent the mean locations of MJO in CP-type ENSO seasons (when RMM amplitude > 1.0) in the RMM1-RMM2 plane. The red, green, blue, and cyan dots represent the mean RMM amplitudes and phase locations during CP-type ENSO MAM, JJA, SON, and DJF. We stratified the data season-wise to identify the exact seasons, showing the MJO frequency patterns during ENSO phases. The black cross mark represents the mean location considering all the seasons. We can observe that during cold CP-type ENSO phases (especially in DJF and SON and except JJA, SON), the mean MJO state is along the positive RMM1 axis, denoting anomalous MJO convection in either of the phase regions 3,4 5 and 6 and anomalous suppression of convection over MJO phase 1,2,7 and 8 (Fig. 7b). This fact is also visible in the MJO frequency anomaly pattern during negative CP-type ENSO seasons (Fig. 3c). However, during the warm CP-type ENSO (especially MAM, JJA and SON except for DJF), the mean position is along the negative RMM1 axis with a small amplitude (Fig. 7a).

Similarly, in Fig. 7c,d the mean location of MJO during the warm EP-type ENSO phases are represented in the RMM1-RMM2 plane. We observe that the mean location of MJO is along the negative RMM2 axis (especially in MAM, JJA, SON except for DJF (Fig. 7c). It denotes anomalous MJO convection in phase locations 1, 2, 3, and 4 and anomalous suppression of convection over MJO phase 5, 6, 7 and 8. This pattern is also visible in the MJO frequency anomalies during warm EP ENSO seasons (Fig. 3b). During cold EP-type ENSO, the mean location of the MJO is along the positive RMM2 axis (especially in DJF, MAM, SON except during JJA) (Fig. 7d). However, we should remember that MJO frequency patterns fully get established after one season of the peak of ENSO.

## Discussion

The relationship between the MJO and ENSO has been studied rigorously in the past few decades where many aspects of their inter-relationship has been revealed. Interannual variability of the MJO can be quantified in terms of the frequency of occurrence of the MJO phases. Interannual variability of MJO frequency at the eight MJO phases (MJO frequency) represent the information on the spatial variability of the MJO. The MJO activity may vary over the warm pool region in a season. Barrier effect is one of the examples of this type of variation where the MJO do not propagate through the Maritime Continent and dissipate over the region. In that case, the number of MJO convective days over the eastern Indian Ocean becomes different from the Western Pacific Ocean due to the lack of MJO propagation beyond the Maritime Continent.

This idea of spatial asymmetry of the MJO activity and its interannual variability leads us to investigate the interannual variability of the MJO frequency over different phase regions. In the present study, we have used the EOF technique on the MJO frequency anomaly data for 156 seasons from 1979 to 2018. We find that there are two dominant spatial modes of MJO frequency anomaly which have significant interannual variation (Fig. 1a,b). The EOF1 and EOF2 of MJO frequency explain almost half of the interannual variability of MJO frequency. The first pattern explains a higher frequency of MJO activity over the Maritime Continent and a lower frequency over the central Pacific Ocean and the western Indian Ocean, or vice versa. The second pattern is associated with a higher frequency of MJO active days over the eastern Indian Ocean and a lower frequency over the western Pacific, or vice versa. We find that these spatial modes are structurally similar and significantly correlated to the Wheeler and Hendon (2004) RMM1 and RMM2 spatial patterns. This suggests that interannual variation of MJO activity follows the WH04 RMM spatial patterns.

Now the question arises, what are the drivers of these two MJO frequency patterns? The drivers could be the tropical SST or large-scale circulations, or it could be due to the internal dynamics of the atmosphere. The role of tropical SST on these two modes is investigated further by observing the linear correlation between the time series of the EOF modes and seasonal average tropical SST conditions (Fig. 1e–h). It is observed that the first EOF mode is significantly correlated to CP ENSO state (with correlation value 0.24 significant at 95% confidence level) whereas the second spatial mode is significantly correlated to canonical ENSO state (with correlation value 0.47 significant at 95% confidence level). Further the lead-lag correlation analysis suggests that these two MJO frequency modes lags the underlying SST condition by one season.

Since, the correlation values are not large, so it is not right to jump into the conclusion that the two ENSO modes are responsible for the two respective MJO frequency EOFs. We, therefore, conducted a composite analysis of MJO frequency anomaly during positive and negative phases of central Pacific and eastern Pacific ENSO seasons. A major association of the MJO frequency with these two types of ENSO modes should make the composites of MJO frequency anomaly look like the two MJO frequency EOF patterns. The composites of MJO frequency anomaly during the central Pacific and eastern Pacific ENSO seasons are similar to MJO frequency EOF1 and EOF2 respectively, suggesting that there is a significant relationship between the MJO frequency EOFs and ENSO modes. So, in summary, the interannual variation of MJO activity follows the WH04 RMM spatial patterns and these patterns are significantly related to two types (CP-type and EP-type) of ENSO phases.

Hence, we tried to understand the interannual variability of the MJO frequency in terms of the two leading EOFs of MJO frequency through the biplot technique (Supplementary Fig. 3 and Fig. 3g,h). Basically, this representation is the scatter plot in two-dimensional leading EOF plane. MJO frequency anomalies for each of the 156 seasons is represented as a position vector in two dimensional EOF plane. The combination of the MJO frequency modes represents the estimate of frequency anomaly in a particular season. We drew data ellipses enclosing the MJO frequency anomaly during central Pacific and canonical ENSO positive and negative states. We find that from the central Pacific cold to warm ENSO state, the mean MJO frequency pattern changes following the MJO frequency EOF1 (WH04 RMM1 pattern), which means that over phase 4, 5, 6 (Maritime Continent) region, the MJO frequency becomes less and over phase region 1, 7 and 8 (central Pacific and the western Indian Ocean) MJO frequency becomes more. From the canonical eastern Pacific cold to warm ENSO phases, MJO frequency anomaly changes following the MJO frequency EOF2 (WH04 RMM2 pattern) pattern which is associated with the increased MJO frequency in the phase regions 2, 3, 4 (central and east Indian ocean) and decreased frequency over phase regions 6, 7, 8 (west to the central Pacific Ocean). From a mathematical point of view, it can be stated that MJO frequency EOFs are observed in MJO frequency data due to the basic MJO frequency state change over the eight phase regions during the two types of ENSO.

From the cold to the warm phase of CP-type ENSO, the mean location of MJO in the RMM1-RMM2 plane changes following the WH04 RMM1 axis (Fig. 7a,b). For the EP-type ENSO, the mean location of MJO in the RMM1-RMM2 plane changes following the WH04 RMM2 axis (Fig. 7c,d). We see that the first MJO frequency pattern is most prominent during the negative central-Pacific (CP-type) ENSO phases (especially during September–November and December-February) when the mean location of MJO is far in the RMM1 direction in the RMM1-RMM2 plane (Fig. 7b). The second pattern is most evident during the positive eastern-Pacific (EP-type) ENSO phases (during March–May, June–September and September–November) when the mean location of MJO is far in the RMM2 direction in the RMM1-RMM2 plane (Fig. 7c). We conducted the composite analyses of different atmospheric variables during CP and EP-type ENSO phases to identify the reason behind MJO frequency spatial patterns. We find that the mean Walker circulation changes during these two types of ENSO phases alter the mean moisture distribution over the equatorial region. The change in the moisture distribution impacts the intraseasonal moist static energy tendencies which restrict the MJO propagation over the different part of the warm pool introducing the zonal asymmetry in MJO propagation. From the intraseasonal moisture budget analysis, we tried to figure out the source of intraseasonal moisture tendency. We find that during the CP-type and EP-type ENSOs, the mean moisture distribution and its convergence through intraseasonal winds are responsible for the intraseasonal moisture tendency and thereby the anomalous MJO frequency pattern.

Previous studies on the interannual variability of MJO frequency were mostly confined to studying the MJO frequency during boreal or extended boreal winter seasons. On the contrary, the current study investigates the MJO frequency for all seasons and tries to identify dominant spatial patterns of MJO frequency which have prominent interannual variability. RMM index data for MJO is calculated from OLR and zonal wind by removing the ENSO signal from the data. We show that the intrinsic influences of ENSO are still present in the RMM MJO frequency data that is separated from the ENSO signal.

## Methods

### ENSO seasons

We computed two type of ENSO indices during the period 1979 to 2018 from Niño 3 and Niño 4 indices following Sullivan et al.^37^. Sullivan et al.^37^ derived EP and CP index from Niño 3 and Niño 4 indices using the following formulas, $EP = Ni\tilde{n} o3\_normalized- 0.5 * Ni\tilde{n} o4\_normalized$ and $CP = Ni\tilde{n} o4\_normalized- 0.5 * Ni\tilde{n} o3\_normalized$. We prepared a seasonal EP and CP time series from the monthly values for four separated seasons, i.e. boreal winter (December–January–February or DJF), boreal spring (March–April–May or MAM), boreal summer (June–July–August or JJA) and boreal autumn (September–October–November or SON). We chose these four seasons considering the evolution of El Niño. El Niño generally evolves from its initial stage during boreal summer (JJA) to its most active stage during boreal winter (DJF) and decays in following boreal spring (MAM). We identified the warm and cold EP-type ENSO seasons when the seasonal time series of the EP index crossed its positive and negative standard deviation values respectively (Supplementary Fig. S5). Thus from 1979 (DJF) to 2018 (SON), positive, negative and neutral EP-type ENSO seasons were identified. Similarly, as EP-type ENSO seasons, warm, cold and neutral phases of CP-type ENSO (El Niño Modoki) were identified based on CP index (Supplementary Fig. S5 and Supplementary Table S2). We identified two types of ENSO phases to examine their influence on MJO variability.

### MJO frequency

The interannual variability of the MJO is investigated in terms of the frequency of occurrence of the MJO phases in boreal winter by numerous studies^28,29,38^. We have adopted the same definition for the frequency of occurrence of MJO phases as in the earlier studies. For convenience, we have abbreviated the term “frequency of occurrence of MJO phases” as MJO frequency in this study. MJO frequency represents the number of MJO active days (with RMM amplitude greater than 1.0) over any particular phase locations in a season. RMM amplitude greater than 1.0, conventionally represent the active MJO state^18^. We calculate MJO frequency at eight RMM phase locations over the time period 1979 to 2018 for DJF, MAM, JJA and SON seasons. From December 1979 to November 2018, MJO frequencies in 156 seasons are considered in this study. This represents the spatial variation of MJO activity throughout the year. For DJF, MAM, JJA and SON, the mean and standard deviation of MJO frequency over the eight phase locations are represented in the Supplementary Figure S6, where we can see the seasonality in MJO frequency data.

### Interannual variation of MJO frequency

MJO frequency possesses seasonal characteristics which we discussed in the introduction section. We removed the seasonality from the MJO frequency data by standardizing each specific season’s data by that particular season’s climatology (e.g. MJO frequency in SON is standardized by SON climatology of MJO frequency) and thus we obtained normalized MJO frequency anomaly data at each phase location (Supplementary Fig. S1a). This normalized MJO frequency anomaly is independent to seasonal characteristics of MJO which are evident through more MJO frequencies in boreal winter and spring than in boreal summer and autumn. The MJO frequency anomaly, therefore, represents the interannual variability of MJO frequency excluding the seasonal cycle of the MJO.

The derived MJO frequency anomaly data is a multivariate dataset which has eight variables representing eight RMM phase regions (m = 8) and 156 cases (n = 156) representing 156 seasons (Supplementary Fig. S1a). We performed multivariate Principal Component analysis (PCA) or Empirical Orthogonal Function analysis (EOF) on this data to explore linearly correlated phase regions having similar interannual MJO frequency variation. We obtain the spatial patterns of MJO frequency variation through PCA in terms of spatial EOFs.

We further use the biplot technique to represent the PCA result in the two-dimensional leading principal component plane. Biplot is not commonly used in the field of meteorology. We discussed the details of PCA and the biplot technique in the following section. Each season was represented in biplot plane according to scores (principal components) of the two EOFs. We further use the concept of data ellipse to enclose the ENSO season points and to describe the statistics of MJO frequency during the ENSO phases. The details of data ellipse are also described in the following section.

### PCA

The basic idea of PCA is to rotate the reference axis of the variables towards the direction of maximum variability in the data. The leading eigenvectors (Empirical orthogonal Functions-EOFs or loadings or principal axis) point towards directions of maximum variability. Structure of the eigenvectors in terms of variables (EOFs) represents the linear relationship between the variables in the direction of maximum variability. The data represented only by dominant eigenvectors are the dimension reduced version of the data explaining a percentage of total variability. The eigenvalues denote the proportion of variance concerning total variance explained by corresponding eigenvectors. The criteria for degeneracy of eigenvalues are discussed by North et al.^32^.

### PCA biplot

In two-dimensional space of two leading principal axes, we used the biplot technique to describe the covariance PCA result. The article Gabriel^39^ is the original foundation of the biplot technique. Jolliffe^36^ discussed the basic concepts of biplot. Using the biplot technique, Takahasi et al.^40^ described two types of ENSO events. Ivanov and Evtimov^41^ used the biplot method on northern hemispheric monthly temperature anomaly data and had explained different attributes of the technique.

Biplot is the most compressed geometrical representation of information from a data matrix, where the attempt is to represent both observation and variables in a two-dimensional space. Covariance biplot describes the covariance PCA outcome in two leading principal component space. Two-dimensional biplot retains first two eigenvectors to give an approximate representation of the data. We will discuss three main basic features of biplot which are its axes, arrows and points. Above mentioned studies explained the detailed theoretical development of these features. The two axes in biplot represent the first two principal axes (EOFs) normalized to unit length by corresponding eigenvalues or variances. The arrow vectors describe the variables in two principal axes space. The length of an arrow represents the standard deviation of the corresponding variable and cosine between two arrows represent the linear correlation between the variables. The position vectors or points denotes the case entry in the centered data matrix. The position vectors are the scoring values (Principal Components-PCs) corresponding to the first two eigenvectors having normalized unit variance. The Euclidean distance between two points denotes the ‘Mahalanobis distance’ between the cases. The ‘Mahalanobis distance’ explains the statistical similarity between the two events. Two cases are statistically similar when they are closer to each other. Therefore, the similar events having less ‘Mahalanobis distance’ form clusters in two-dimensional biplot. The limitations come in biplot when the number of variables is high, and difficulty arises to distinguish between variable vectors. However, biplot allows visual appraisal of the inherent structure of the data, its variance and covariance structures, clustering of the events, extremes and multivariate outliers.

### Clusters and data ellipse

To represent the clusters of events in biplot space we used the data or concentration ellipse^42^. The data ellipse represents a visual summary of a scatter plot indicating the means, standard deviations, correlation, and the slope of the regression line for two variables^43^. Friendly et al.^43^ discussed the role of ellipsoids in statistical data analysis and visualizations. For a bivariate normally distributed data $x = \left( {x1,x2} \right)$, probability density function $\phi \left( x \right)$ is given by

1 $$\phi \left( x \right) = 1/2\pi \left| \Sigma \right|^{ - 1/2} exp\left\{ { - 1/2\left( {x - \mu } \right)^{{\prime }} \Sigma^{ - 1} \left( {x - \mu } \right)} \right\},\;where\;\Sigma = \left( {\sigma_{11} \;\sigma_{12} \; \sigma_{21} \; \sigma_{22} } \right)$$

Here $$\Sigma$$ is the covariance matrix of bivariate normal data $$x$$. The quadratic form in the exponent of the equation is a statistical distance measure, often referred to as Mahalanobis distance. Mahalanobis distance is the squared statistical distance of $$x$$ from $$\mu$$ accounting the fact that the variables may be correlated and have different variances. The quadratic form in the exponent follows the $$\chi^{2}$$ distribution with two degrees of freedom. Constant density contours of bivariate normally distributed data follow the equation of an ellipse with $$\varepsilon_{c} = \left( {x - \mu } \right)^{{\prime }} \Sigma^{ - 1} \left( {x - \mu } \right) = c^{2}$$, where c is the size of the ellipse. The ellipse is generally referred to as data or concentration ellipse. The data ellipse encloses the points having squared Mahalanobis distance $$D^{2} < = c2 = \chi^{2} \left( \alpha \right)$$. One standard deviation data ellipse encloses 68% of the data with $$c^{2} = \chi^{2} \left( {\alpha = 0.32} \right) = 2.28$$. Where 95% data ellipse has $$c^{2} = \chi^{2} \left( {\alpha = 0.05} \right) = 5.99$$. The axes of the ellipse are in the direction of the eigenvectors of the covariance matrix $$\Sigma$$ and the length of the axes are proportional to $$\sqrt {\lambda_{1} }$$ and $$\sqrt {\lambda_{2} }$$. The orientation of the semi-major axis ($$\lambda_{1}$$) of an ellipse with the reference axes depicts the positive or negative correlation between the two normally distributed variables. The area of the data ellipse containing the central $$\left( {1 - \alpha } \right) \times 100\%$$ of a bivariate normal data is $$\pi c^{2} \sqrt {\sigma_{11} \sigma_{22} \left( {1 - \rho^{2} } \right)}$$ . The eccentricity of a data ellipse is high ($$\lambda_{1} > \lambda_{2}$$) when there is a large variance in one variable ($$\sigma_{11} > \sigma_{22}$$ or opposite) or significant covariance existed ($$\sigma 12$$) between two variables. A low eccentricity indicates similar variances ($$\sigma_{11} \approx \sigma_{22}$$) and less covariance (independent variables with $$\sigma_{12} \approx 
0$$) among the variables. For PCA, biplot and data ellipse, we have used two R library, “FactoMineR" and “factoextra"^44^.

### Intraseasonal parameters

We applied a 20–100 days band-pass filter on daily OLR (a proxy of convection) to extract the intraseasonal signal of OLR. We compute the seasonal mean of the filtered OLR anomaly for DJF, MAM, JJA, SON seasons from 1979 to 2018. We obtained the space time filtered (1–10 wavenumber and 20–100 days periodicity) CMAP daily precipitation data representing eastward propagating MJO signal^45^. We also computed the seasonal mean SST, omega (ω), specific humidity (q) anomaly from the monthly datasets.

Maloney^46^ explained that the intra-seasonal moist static energy (MSE) budget could describe the eastward propagation of MJO. The recharge of column integrated MSE at intra-seasonal timescale occurs at the east of the MJO convection center with the help of low-level easterlies and mean background moisture field which helps MJO to propagate eastward. The discharge of column integrated MSE occurs during and after the precipitation occurs at MJO convection along with the lower level westerly anomalies, which stops MJO movement in the westward direction. Therefore, MSE tendency is positive at the east side of MJO convection center and negative at the west side (behind) it. With the help of the moist static energy and its tendency, we tried to explain the result in our study. We computed the moist static energy ($m = C_{p} T + Lq + {\varphi}$) from air temperature, specific humidity and geo-potential height dataset. Then, we obtained the intraseasonal (20–100 days filtered) moist static energy anomaly ($m^{{\prime }}$) and moist static energy tendency $\left\langle {dm^{{\prime }} /dt} \right\rangle$.

### Intraseasonal moisture budget

To investigate the moisture budget at intraseasonal scale, we examine the moisture budget at intraseasonal time scale Eq. (2). In left hand side of Eq. 4, the term denotes the intraseasonal moisture tendency. At right hand side, the first time denotes the horizontal moisture advection, the second term explains the horizontal moisture convergence. The third term depicts the flux from vertical moisture advection and fourth terms denotes the apparent moisture sink^47^ .The first two term denotes the intra-seasonal moisture flux convergence. Second and third term together denotes the vertical moisture advection.

2 $$\frac{{\partial q^{{\prime }} }}{\partial t} = - \left( {V \cdot \nabla q} \right)^{{\prime }} - \left( {q\nabla \cdot V} \right)^{{\prime }} - \left( {\frac{{\partial {\upomega }q}}{\partial p}} \right)^{{\prime }} + \frac{{Q_{2}^{{\prime }} }}{L}$$

To understand the eddy-eddy, eddy-mean flow interaction we decomposed each field in low frequency background state (> 100 days; overbar; LFBS), intraseasonal (20–100 days; prime; MJO) and a high frequency (< 20 days; asterisk; synoptic) part according to Eq. (3).

3 $$q = \overline{q} + q^{{\prime }} + q^{*}$$

Following Hsu and Li.^48^, the horizontal moisture convergence can be further decomposed into four part following the Eq. (4). We further decomposed the horizontal moisture convergence term in these four terms to investigate the eddy-eddy, eddy-mean flow interaction.

4 $$- \left( {q\nabla \cdot V} \right)^{{\prime }} = - { }\left( {\overline{q}\nabla \cdot V^{{\prime }} } \right)^{{\prime }} - \left( {q^{{\prime }} \nabla \cdot \overline{V}} \right)^{{\prime }} - \left( {q^{{\prime }} \nabla \cdot V^{{\prime }} } \right)^{{\prime }} - \left( {q^{*} \nabla \cdot V^{*} } \right)^{{\prime }}$$

Here, all the terms have been vertically integrated from 1000 to 300 hPa. We specifically focused on terms in Eq. (4) related to intraseasonal moisture convergence.

## Supplementary Information

Supplementary Information.

## Data Availability

The present study is based on the period 1979 to 2018, corresponding to the availability of OLR and the RMM index in the satellite era^5^. RMM index data from 1979 to 2018 is obtained from the Australian Bureau of Meteorology. We have lowpass filtered the RMM index with a cut-off period of 10 days to avoid high frequency variability. Monthly Niño 3 and Niño 4 indices are calculated from Extended Reconstructed Sea Surface Temperature version 5 (ERSSTv5) dataset with 2 × 2 resolution^49^. These indices are used to identify the warm and cold Eastern Pacific (EP)‐type and central Pacific (CP)-type ENSO phases^35,50^. We used daily NOAA interpolated OLR data with 2.5 × 2.5 resolution in our present study^51^. We obtained the 2.5 × 2.5 pentad CPC Merged Analysis of Precipitation (CMAP) data and interpolated the data to daily^52^. We used the zonal wind (u), meridional wind (v), omega (ω), specific humidity (q), air temperature (T) and geopotential height (φ) from NCEP/NCAR reanalysis 1 dataset^53^. We also used monthly Hadley Centre Sea Ice and Sea Surface Temperature data set (HadISST) to check the consistency of our results^54^.
