# Author Correction: Interannual variability of the frequency of MJO phases and its association with two types of ENSO

**DOI:** 10.1038/s41598-021-93447-7

**Published:** 2021-07-15

**Authors:** Panini Dasgupta, M. K. Roxy, Rajib Chattopadhyay, C. V. Naidu, Abirlal Metya

**Affiliations:** 1 Indian Institute of Tropical Meteorology, Ministry of Earth Sciences, Pune, 411008 India; 2 Department of Meteorology and Oceanography, College of Science and Technology, Andhra University, Visakhapatnam, Andhra Pradesh 530003 India; 3 India Meteorological Department, Ministry of Earth Sciences, Pune, 411005 India; 4 Department of Atmospheric and Space Sciences, Savitribai Phule Pune University, Pune, 411007 India

Correction to: *Scientific Reports* 10.1038/s41598-021-91060-2, published online 02 June 2021

The original version of this Article contained errors in Figure 5, where the x-axis labels ‘Longitude’ were truncated in both graphs.

The original Figure  5 and its accompanying legend appear below.

**Figure 5:**
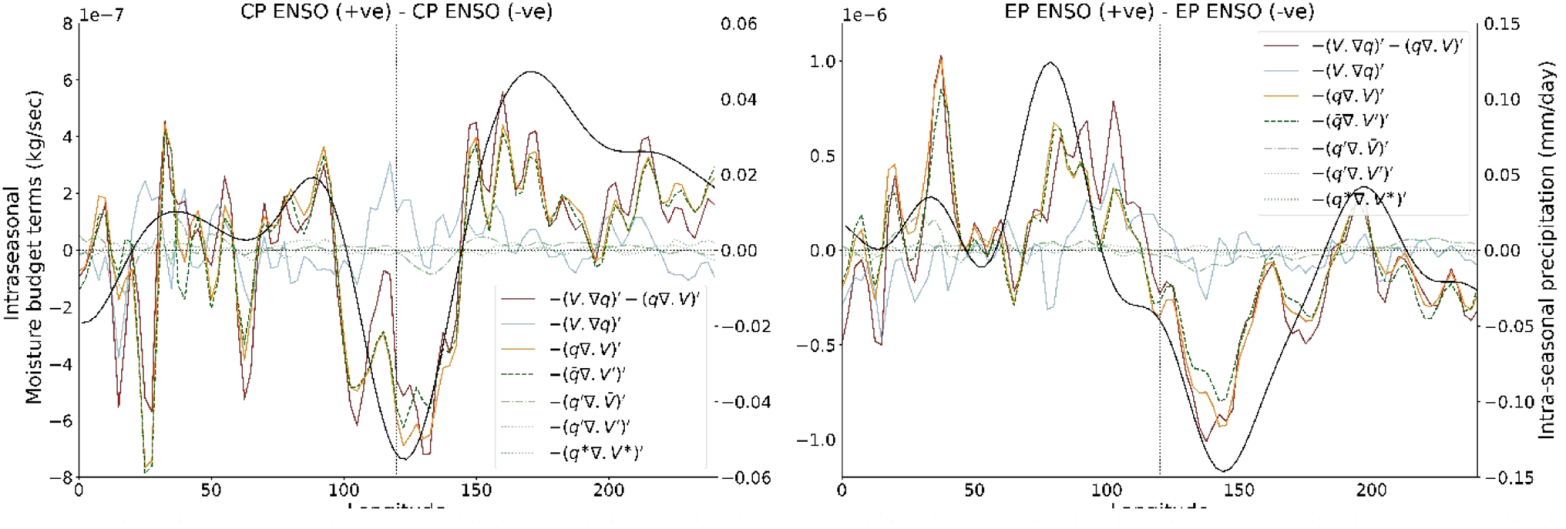
Differences in moisture budget terms in Eqs. (2 and 4) between the warm and cold CP-type ENSO phases (left panel). The red line denotes the horizontal moisture flux convergence [sum of horizontal advection (blue) and moisture convergence (yellow)]. The green lines are the four terms of horizontal moisture convergence in Eq. (4). We observed that the dashed green line denoting the convergence of mean moisture through intraseasonal winds have the largest contribution in intra seasonal moisture tendencies (vertical advection due to horizontal moisture convergence). The right panel shows the difference in moisture budget terms for warm and cold EP-type ENSO phases.

The original Article has been corrected.

