# Supplementary material for: Interannual variability of the frequency of MJO phases and its association with two types of ENSO
Source: Sci Rep. 2021 Jun 2;11:11541. doi: 10.1038/s41598-021-91060-2 (PMC8172938; doi:10.1038/s41598-021-91060-2)
Supplement: Supplementary file 1 — Supplementary Information. [file 41598_2021_91060_MOESM1_ESM.docx]

Interannual variability of the frequency of MJO phases and its association with two types of ENSO

**Panini Dasgupta**^1,2*^, **M K Roxy**^1^**, Rajib Chattopadhyay**^3^**, C. V. Naidu**^2^, **Abirlal Metya**^1,4^

*^1^ Centre for Climate Change Research, Indian Institute of Tropical Meteorology, Ministry of Earth Sciences, Pune 411008, India*

^2^*Department of Meteorology and Oceanography, College of Science & Technology, Andhra University, Visakhapatnam, Andhra Pradesh 530003, India*

*^3^Indian Institute of Tropical Meteorology, Ministry of Earth Sciences, Pune 411008, India*

*^4^Department of Atmospheric and Space Sciences, Savitribai Phule Pune University, Pune, India*

*^*^Correspondence to panini.dasgupta@tropmet.res.in*

## Supplementary Figures


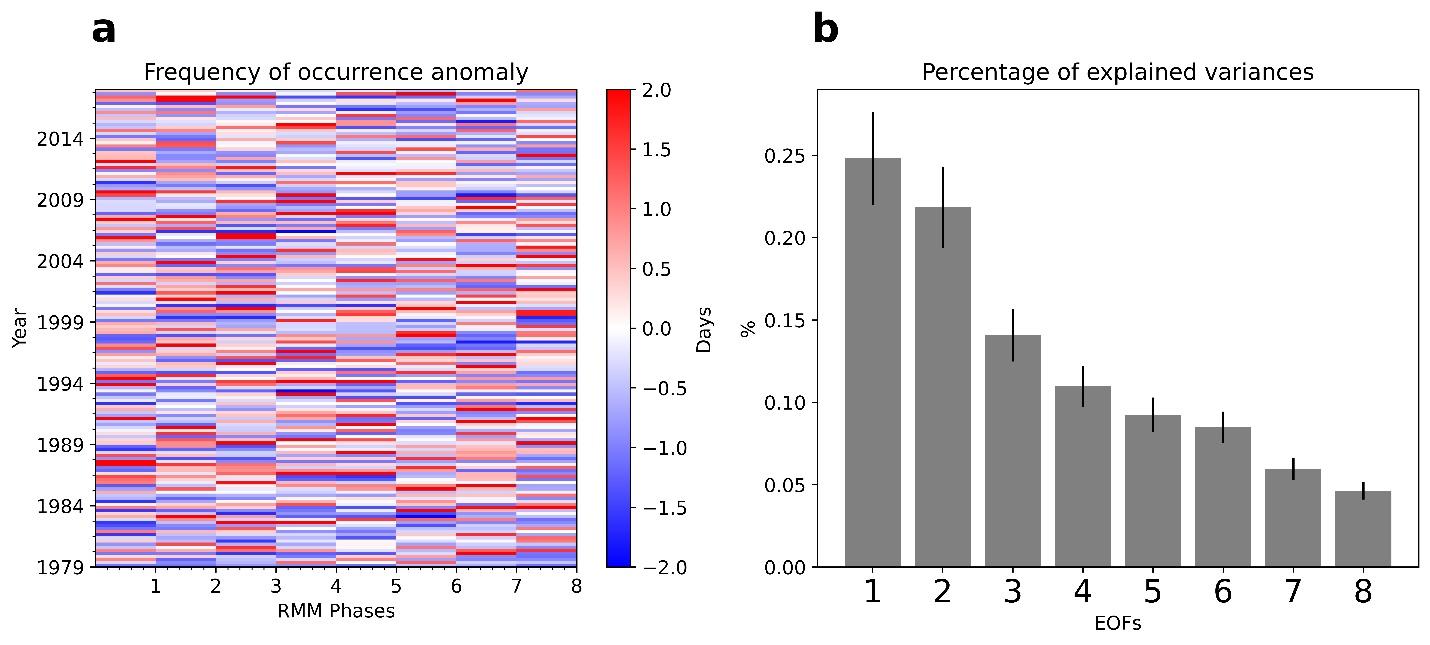


**Supplementary Figure S1.** **(a)**MJO frequency anomaly (seasonally normalized) at eight RMM MJO phase locations (days), **(b)** Scree plot denoting the percentage of variance contributed by the eight eigenvectors. The error bars indicate the North et al.^1^ criteria for the EOFs.


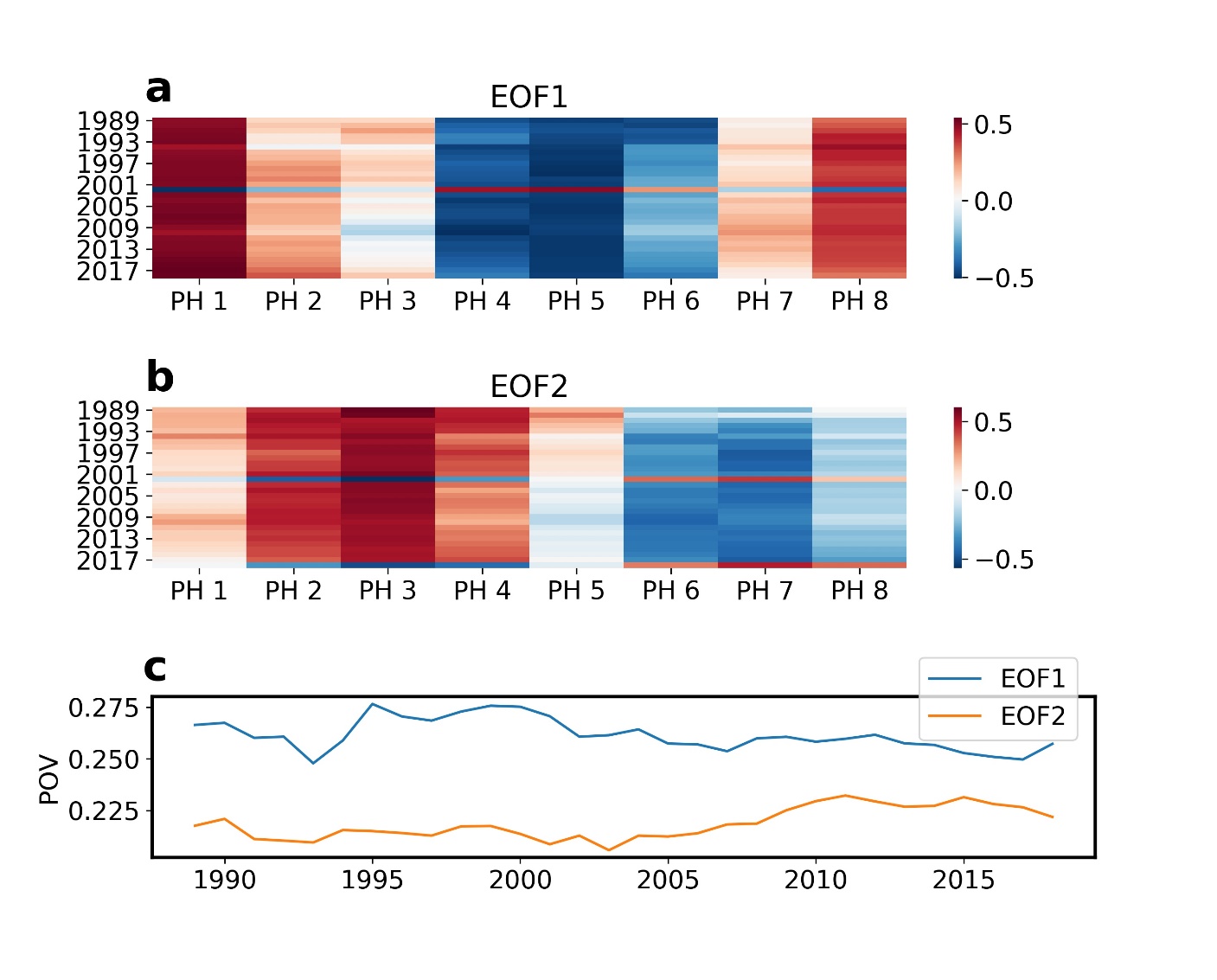


**Supplementary Figure S2.** **(a)** Time evolution of EOF1 of MJO frequency from 1979-1989 to 1979-2018. **(b)** Time evolution of EOF2 of MJO frequency from 1979-1989 to 1979-2018. **(c)** Time evolution of percentage of variance explained by EOF1 and EOF2 from 1979-1989 to 1979-2018.


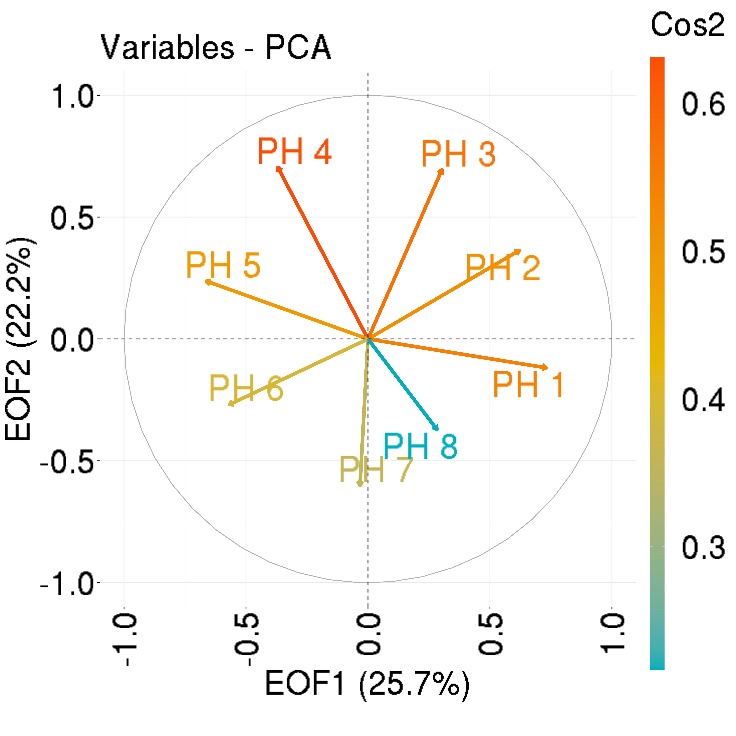


**Supplementary Figure S3:** PCA-Biplot is denoting the relationship among the MJO frequencies at the eight RMM phase locations. The color scale implies the cos2 (percentage of variance explained in EOF1-EOF2 space) of the MJO frequency.


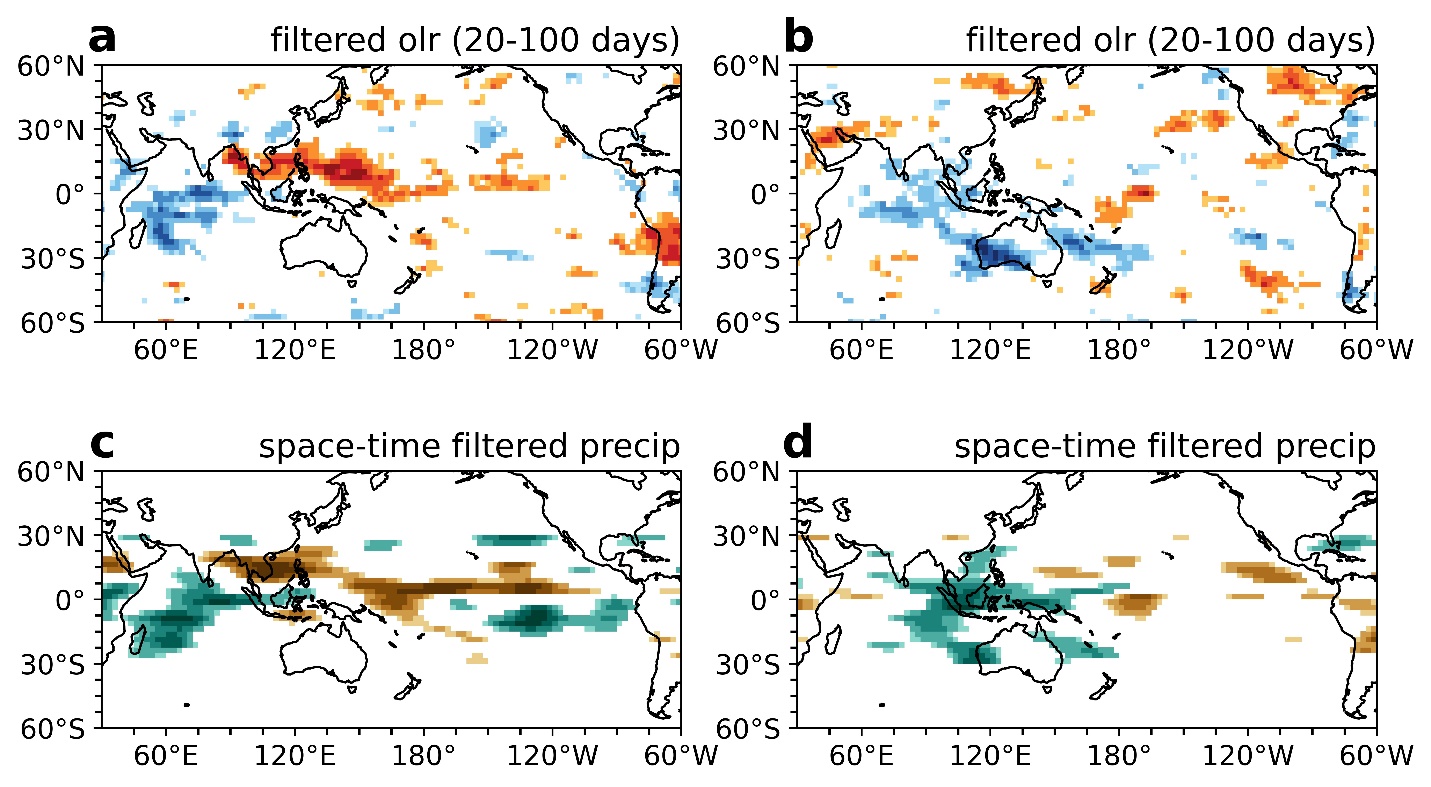


**Supplementary Figure S4. (a) and (b)** MJO frequency EOF1 and EOF2 related 20-100 days filtered seasonal mean OLR (W/m^2^) anomaly. Similarly, **(c) and (d).** represent 20-100 days and wavenumber 1–10 space-time filtered precipitation anomaly (mm/day). Only the correlation values exceeding 90% confidence level based on the Student's t test are represented. MJO frequency EOF time-series has four data points per year (DJF, MAM, JJA, SON); from 1979-2018 total of 156 data points. We regressed the two EOF timeseries with three monthly mean OLR/precipitation anomalies (daily) to extract the spatial patterns.


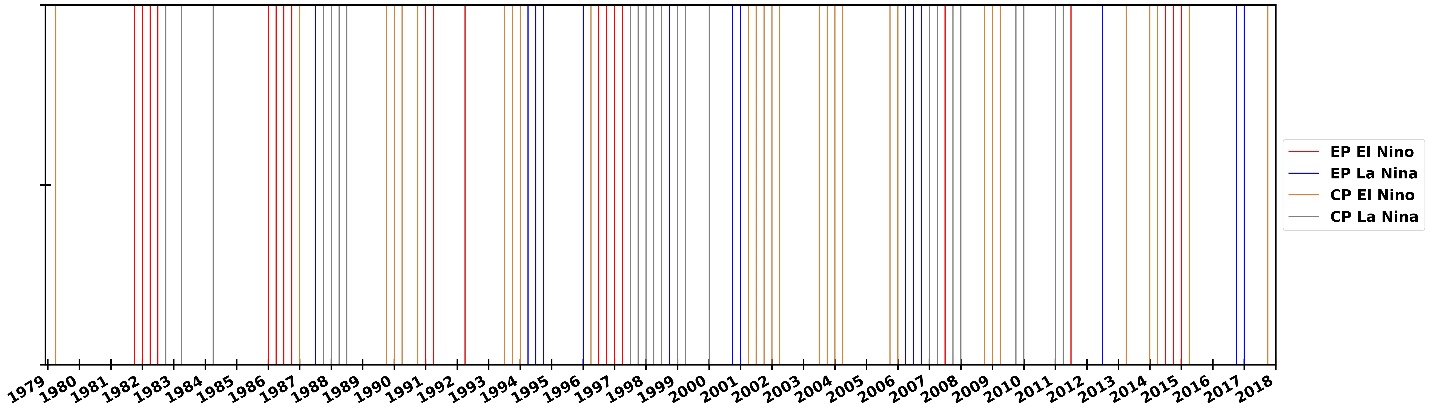


**Supplementary Figure S5**. CP type and EP type ENSO seasons from 1979-2018. Each bar represents a three-month season (i.e., DJF, MAM, JJA, SON).


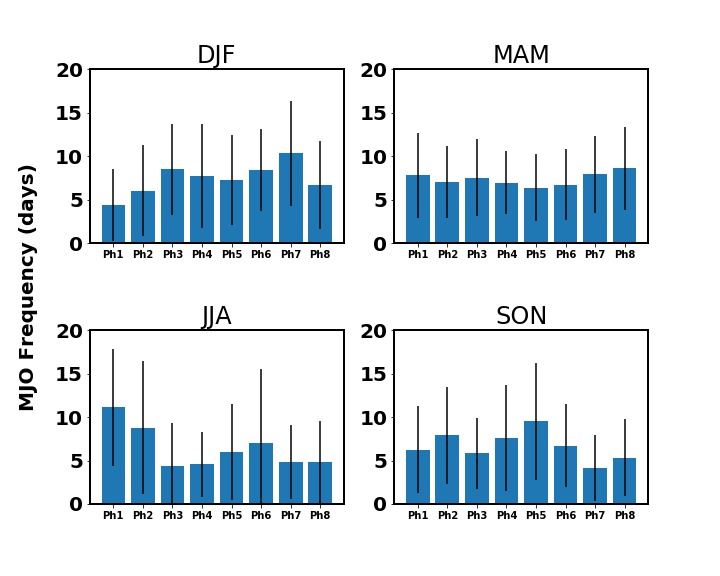


**Supplementary Figure S6**. Mean and standard deviation of MJO frequency in DJF, MAM, JJA and SON.

**Supplementary Table S1:** Correlation coefficients and cos2 between MJO frequency anomalies at eight RMM phase locations and MJO frequency EOF timeseries.

| **Correlation** | **PH1** | **PH2** | **PH3** | **PH4** | **PH5** | **PH6** | **PH7** | **PH8** |
| --- | --- | --- | --- | --- | --- | --- | --- | --- |
| **PC1** | 0.76 | 0.50 | 0.22 | -0.49 | -0.68 | -0.51 | 0.08 | 0.42 |
| **PC2** | -0.01 | 0.43 | 0.66 | 0.56 | 0.06 | -0.42 | -0.64 | -0.47 |
| **Cos2** |  | | | | | | | |
| **PC1** | 0.59 | 0.24 | 0.05 | 0.24 | 0.46 | 0.26 | 0.01 | 0.18 |
| **PC2** | 0.00 | 0.18 | 0.44 | 0.32 | 0.00 | 0.18 | 0.41 | 0.22 |

**Supplementary Table S2:** List of CP type and EP type ENSO seasons from 1979-2018.

| **Positive CP ENSO** | **Negative CP ENSO** | **Positive EP ENSO** | **Negative EP ENSO** |
| --- | --- | --- | --- |
| 1980-MAM,  1987-DJF,  1990-SON,  1990-DJF,  1991-MAM,  1991-SON,  1994-JJA,  1994-SON,  1994-DJF,  1997-MAM,  2002-MAM,  2002-JJA,  2002-SON,  2002-DJF,  2003-MAM,  2004-JJA,  2004-SON,  2004-DJF,  2005-MAM,  2006-SON,  2006-DJF,  2009-SON,  2009-DJF,  2010-MAM,  2014-MAM,  2014-DJF,  2015-MAM,  2016-MAM,  2018-SON | 1983-SON,  1984-MAM,  1985-MAM,  1988-SON,  1988-DJF,  1989-MAM,  1989-JJA,  1998-JJA,  1998-SON,  1998-DJF,  1999-MAM,  1999-JJA,  1999-DJF,  2000-MAM,  2000-DJF,  2007-DJF,  2008-MAM,  2008-SON,  2008-DJF,  2010-SON,  2010-DJF,  2011-DJF,  2012-MAM | 1982-SON,  1982-DJF,  1983-MAM,  1983-JJA,  1986-DJF,  1987-MAM,  1987-JJA,  1987-SON,  1991-DJF,  1992-MAM,  1993-MAM,  1997-JJA,  1997-SON,  1997-DJF,  1998-MAM,  2008-JJA,  2012-JJA,  2015-JJA,  2015-SON,  2015-DJF | 1988-JJA,  1995-MAM,  1995-JJA,  1995-SON,  1996-DJF,  1999-SON,  2001-SON,  2001-DJF,  2007-MAM,  2007-JJA,  2007-SON,  2013-JJA,  2017-SON,  2017-DJF |
